# Neural Correlates of Decision-Making Under Ambiguity and Conflict

**DOI:** 10.3389/fnbeh.2015.00325

**Published:** 2015-11-27

**Authors:** Helen Pushkarskaya, Michael Smithson, Jane E. Joseph, Christine Corbly, Ifat Levy

**Affiliations:** ^1^Department of Agricultural Economics, University of KentuckyLexington, KY, USA; ^2^Department of Diagnostic Radiology, Yale School of MedicineNew Haven, CT, USA; ^3^Section of Comparative Medicine, Yale School of MedicineNew Haven, CT, USA; ^4^Research School of Psychology, The Australian National UniversityCanberra, ACT, Australia; ^5^Department of Anatomy and Neurobiology, University of KentuckyLexington, KY, USA; ^6^Department of Neurobiology, Yale School of MedicineNew Haven, CT, USA

**Keywords:** decision making, uncertainty, ambiguity, conflict, fMRI

## Abstract

**HIGHLIGHTS**

- We use a simple gambles design in an fMRI study to compare two conditions: ambiguity and conflict.
- Participants were more conflict averse than ambiguity averse.
- Ambiguity aversion did not correlate with conflict aversion.
- Activation in the medial prefrontal cortex correlated with ambiguity level and ambiguity aversion.
- Activation in the ventral striatum correlated with conflict level and conflict aversion.

Studies of decision making under uncertainty generally focus on imprecise information about outcome probabilities (“ambiguity”). It is not clear, however, whether conflicting information about outcome probabilities affects decision making in the same manner as ambiguity does. Here we combine functional magnetic resonance imaging (fMRI) and a simple gamble design to study this question. In this design the levels of ambiguity and conflict are parametrically varied, and ambiguity and conflict gambles are matched on expected value. Behaviorally, participants avoided conflict more than ambiguity, and attitudes toward ambiguity and conflict did not correlate across participants. Neurally, regional brain activation was differentially modulated by ambiguity level and aversion to ambiguity and by conflict level and aversion to conflict. Activation in the medial prefrontal cortex was correlated with the level of ambiguity and with ambiguity aversion, whereas activation in the ventral striatum was correlated with the level of conflict and with conflict aversion. These novel results indicate that decision makers process imprecise and conflicting information differently, a finding that has important implications for basic and clinical research.

## Introduction

Our ability to make effective decisions is considerably affected by the quality of information we receive. Imagine that you are attending the Kentucky Derby for the first time. You are joined by two friends, both experienced race watchers, with inside information about the race horses. Two horses seem promising, and you ask for your friends' advice in selecting one of these horses to bet on. One of your friends is very confident that horse A's odds are twice as high as those of horse B, while your other friend strongly disagrees and insists that he has solid evidence supporting the exact opposite. How do you make a decision in this case? If you equally trust both of your friends, how would you pick one of the horses over the other one? Or would you, perhaps, pick a third horse just to avoid having to choose between them? Compare this scenario with a different one, in which your friends agree that the likelihoods of both horses to win are equally unclear. In which case are you more comfortable making a choice, and in which case do you trust your friends more? Both of these scenarios involve uncertainty around outcome likelihoods, but the nature of this uncertainty is different in the two scenarios. In the first one, there is a “conflict” between two likelihood estimates. In the second, there is “ambiguity” around the likelihood. The goal of this paper is to compare how people make decisions under these two types of uncertainty, both behaviorally and neurally.

Studies of decision making under uncertainty have provided insights into the neural processing of missing information about outcome probabilities (Platt and Huettel, 2008) and have informed clinicians as to the neurobiological abnormalities that might underlie the symptomatology of anxiety-based diseases (Ernst and Paulus, 2005). Most of these studies have focused on *risk*—a situation with known outcomes and known probabilities of these outcomes (von Neumann and Morgenstern, 1944), and *ambiguity*—a situation with known outcomes and unknown probabilities (Ellsberg, 1961).

Definitions of ambiguity, however, have been somewhat limited and inconsistent. Theoretical studies have defined ambiguity as occurring “when there are questions of reliability and relevance of information, and particularly where there is conflicting opinion and evidence” (Ellsberg, 1961, p. 659); whereas the majority of empirical studies have operationalized *ambiguity* as a situation with *imprecise* information about probabilities. Budescu and Wallsten (1995) pointed out this inconsistency and suggested that people process ambiguous information (a “true” value belongs to some known interval) and conflicting information (two or more precise but divergent messages about a “true” value are available) differently, and that ambiguous and conflicting information have different effects on the choice of decision-making strategies.

Note that the *conflict* in this context is somewhat different from the *decision conflict*, or *cognitive conflict*, that has been extensively researched by cognitive neuroscience. Decision conflict occurs when people feel uncertain as to which option to choose from a set of similarly attractive (or unattractive) options, which can lead to suboptimal decision making (Pochon et al., 2008). That is, decision conflict is typically used synonymously with difficulty of selecting the objectively optimal response in the presence of interference (i.e., noise). It is commonly assumed that with higher cognitive (e.g., attentional, Petersen and Posner, 2012) costs decision conflicts can be resolved, and thus suboptimal outcomes (Pochon et al., 2008) can be avoided. In fact, a number of decision tasks have been used to access individual ability to resolve decision, or cognitive, conflicts, and choose optimally even in the presence of strong interference [the Stroop task, (Stroop, 1935), the Flanker task (Eriksen and Eriksen, 1974), the Attention Network Test (Fan et al., 2002, 2009), and many others]. Conversely, in the example above, additional cognitive effort cannot help in identifying the optimal response; only additional information about your disagreeing friends or two horses' history may be, potentially, helpful. A decision has to be made, however, before this information may be available for resolving the informational conflict. That is, “the efficient mind” has to accept that the optimal response cannot be known, and thus additional cognitive effort under these circumstances is not appropriate. The intuitive similarities and differences between the informational conflict and cognitive conflict are a fruitful research topic for future studies, but beyond the scope of this paper.

So far only a handful of behavioral studies have explored the difference between ambiguity and conflict, but the results have been quite consistent: people tend to avoid conflicting unambiguous messages from two equally believable sources in favor of two informatively equivalent, ambiguous, but agreeing messages from these sources (Smithson, 1999; Cabantous, 2007; Cabantous et al., 2011), but see Baillon et al. (2012) for alternative results. In the context of Kentucky Derby (see above), it implies that most people would rather receive advice from two friends, who both admit not knowing anything about horses (ambiguous agreeing messages) than from two absolutely confident friends, who strongly disagree about the chances of horses A and B (conflicting unambiguous messages).

Conflict aversion is particularly strong for low and high probability events (Baillon and Cabantous, 2009). Furthermore, decision makers tend to perceive sources of conflicting information as “not trustworthy,” but believe that they can trust agreeing sources of ambiguous information (Smithson, 1999). Intriguing results from Bugental et al. (1971) might even suggest that a prolonged exposure to conflicting, but not ambiguous, information might provoke the onset of some anxiety-based disorders.

Nevertheless, most research still treats conflict as identical to ambiguity. We tested this hypothesis using a simple gambles design in conjunction with functional magnetic resonance imaging (fMRI). In this design ambiguity gambles are matched with conflict gambles on expected value. The hypothesized equivalence of ambiguity and conflict predicts that: (1) attitudes toward ambiguity correlate with attitudes toward conflict, (2) a largely overlapping set of brain regions is involved in the processing of ambiguity and conflict, (3) behavioral attitudes toward ambiguity and conflict are predicted by the activation in this largely overlapping set of regions. However, our data reveal persistent differences between how ambiguous and conflicting data are processed. To the best of our knowledge, this is the first study suggesting that *conflicting* information and *ambiguous* information about outcome probabilities have qualitatively different effects on decision making, both behaviorally and neurally.

## Materials and methods

### Participants

Forty-two right-handed participants [21 males, mean age = 24.6 ± 5.2 (STD) years] with normal or corrected-to-normal vision provided written informed consent in accordance with the University of Kentucky Institutional Review Board guidelines, and were compensated for their time. Four participants were excluded from the analysis because of excessive head motion (more than 1.7 mm); data from six participants were lost due to technical problems. Data from 32 participants (16 males, mean age = 25.2 ± 5.6) were included in the analysis.

### Procedure and stimuli

Before the fMRI session, participants were informed that in the scanner they would be asked to play a series of lotteries. For each lottery, they would have to guess the type of card that they would draw from a mixed deck of 100 different cards (e.g., *Trivial Pursuit, Pokémon*, etc.). On each trial, participants could refuse to gamble, in which case they received $3. If they chose to play the lottery, they would receive $10 for a correct guess or nothing for an incorrect guess (thus, the expected value of each lottery was equal to the probability of winning multiplied by 10). Lotteries were played under four different informational conditions: *risk, ignorance, ambiguity* and *conflict*. Results from the first three conditions have been reported elsewhere (Pushkarskaya et al., 2010). Here we focus only on the effects of ambiguity and conflict on decision making. We also include risk in our analyses as a reference condition.

**Figure 1:**
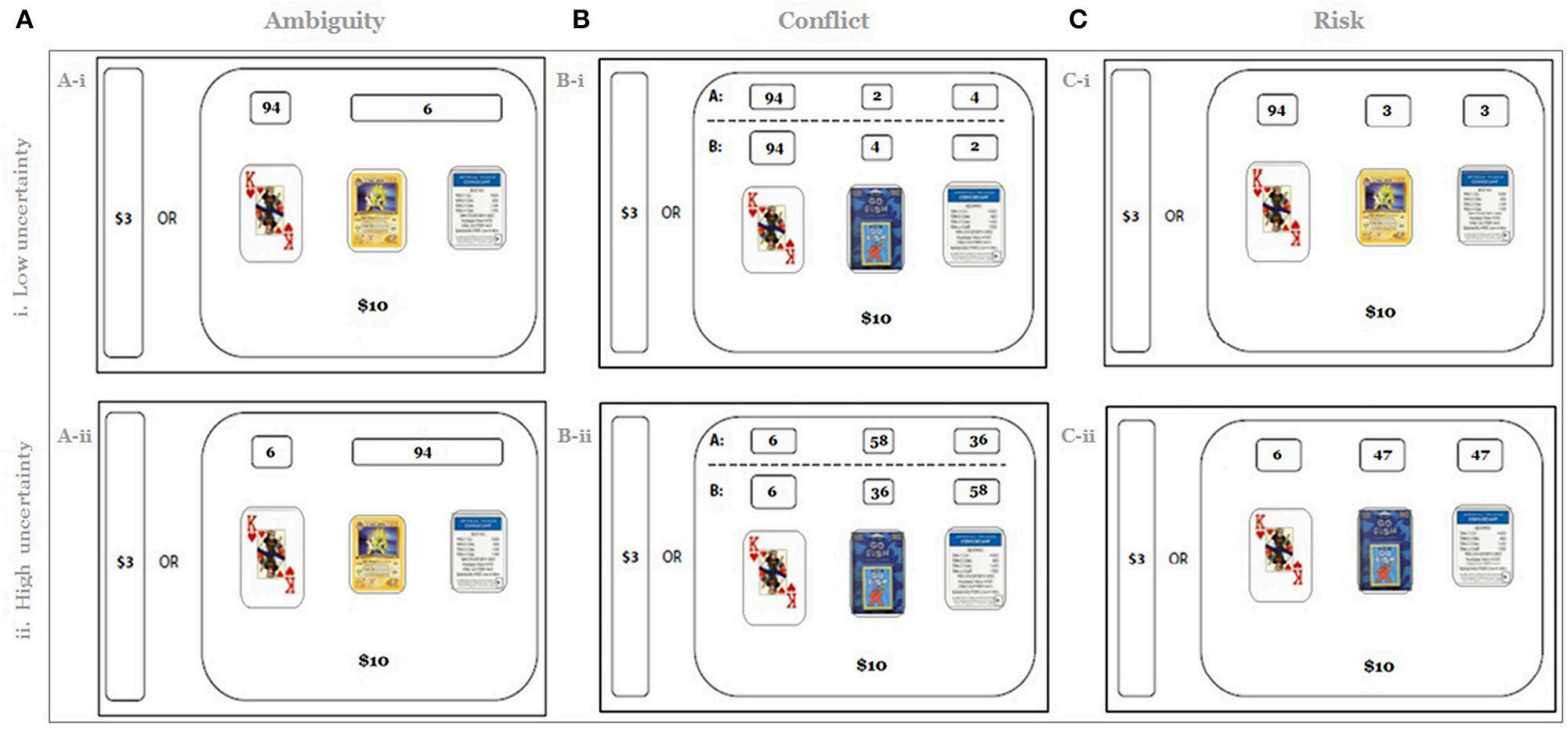
**Experimental design**. Participants were asked to play a series of lotteries under several experimental conditions (**A**: ambiguity, **B**: conflict, **C**: risk). For each lottery, they would have to guess the type of card that they would draw from a mixed deck of 100 different cards (e.g., *Trivial Pursuit, Pokémon*, etc.). On each trial, participants could refuse to gamble, in which case they received $3. If they chose to play the lottery, they would receive $10 for a correct guess or nothing for an incorrect guess. In the ambiguity condition, the total number of “Type 2” and “Type 3” cards was provided (e.g., total of six Pokémon and Monopoly cards, **A-i**). In the conflict condition, participants received conflicting information about the numbers of Type 2 and Type 3 cards (e.g., two Monopoly cards and four Go-Fish cards vs. four Monopoly cards and two Go-Fish cards, **B-i**). In the risk condition the number of cards of Type 2 was always equal to the number of cards of Type 3. Note that the ambiguity gambles **(A)** are matched with the conflict gambles **(B)** and with risk gambles **(C)** on both expected value and variance under the assumption of a rational decision maker. The total number of cards in Piles 2 and 3 in this paper is referred to as the level of ambiguity (conflict); 30 unique combinations of cards of Type 1, Type 2, and Type 3 under each type of uncertainty were included in the experiment (see Supplementary Materials S.1); level of ambiguity (conflict) thus varied from 6 (**A-i**, **B-i**, the lowest of 30 levels of uncertainty) to 94 (**A-ii**, **B-ii**, the highest of 30 levels of uncertainty).

In all three conditions the 100-card mixed deck consisted of cards of three different types. The quantity of “Type 1” cards was always known (e.g., 94 Poker cards, Figure 1, top row); its distribution was the same for all conditions. Since the total number of all cards in the deck was always equal to 100, the distribution of the total number of “Type 2” and “Type 3” cards was also the same across all conditions. However, information about the composition of Type 2 and Type 3 cards varied across conditions (the experimental variable of interest in the study).

In the risk condition, the composition of Type 2 and Type 3 cards was fully known (the number of cards of Type 2 was always equal to the number of cards of Type 3, Figure 1C-i). In the ambiguity condition, only the total number of Type 2 and Type 3 cards was provided (e.g., total of six Pokémon and Monopoly cards, Figure 1A-i), but the composition of Type 2 and Type 3 cards was not. In the conflict condition, participants received conflicting information about the composition of Type 2 and Type 3 cards (e.g., either two Monopoly cards and four Go-Fish cards or four Monopoly cards and two Go-Fish cards, Figure 1B-i). Since the quantity of Type 1 cards was known under all conditions, a risky option was always available to the participants. Therefore, to investigate the effect of ambiguous and conflicting information on decision making in both behavioral and fMRI data analyses we used risk as a reference condition.

Thirty unique gambles were used in each uncertain condition. Two randomly selected gambles from each condition were used twice. The total number of trials in each condition, therefore, was equal to 32. The full list of gambles is reported in Table S.1 (Supplementary Materials). Note that the task calibration has the following four important features. First, the risk, ambiguity, and conflict conditions were matched on expected value. A decision maker, who is not affected by ambiguity or conflict, should therefore treat both the ambiguity and conflict conditions in the same manner as she treats the risk condition. In contrast, an ambiguity (conflict) averse decision maker would bet on cards of Type 2 or Type 3 less often under ambiguity (conflict) than under risk, whereas an ambiguity (conflict) seeking decision maker would bet on cards of Type 2 or Type 3 more often under ambiguity (conflict) than under risk.

Second, in this design, outcome variance under ambiguity was higher than under conflict under the assumption of an uncertainty neutral decision maker. It has been suggested that aversion to outcome variance (Preuschoff et al., 2006) may explain the difference in choices under different types of uncertainty. In our study, a decision maker sensitive to outcome variance should avoid ambiguity more strongly than conflict.

We did not explicitly state in the instructions that all gambles were matched on expected value, and that variance under ambiguity was higher than under conflict, to avoid a possible framing effect. We rather gave the participants a chance to form their own beliefs about the underlying distribution of cards under different conditions. One of the possible explanations of ambiguity (or conflict) aversion is an irrationally pessimistic belief that the unknown odds are always against a decision maker. Arguably, participants' choices can indicate whether these beliefs are affected differently by different types of uncertainty.

Third, to manipulate the level of uncertainty, the total number of Type 2 and Type 3 cards varied from 6 to 94. Thirty unique combinations of cards of Type 1, Type 2, and Type 3 were used in each type of uncertainty (See Supplementary Materials S.1). Figure 1 presents the extreme cases, in which this total number was either 6 (top row, the lowest of 32 levels of uncertainty) or 94 (bottom row, the highest of 32 levels of uncertainty). This manipulation allows varying the level of unknown in ambiguity trials and the level of disagreement in conflict trials in the task. This, in turn, allowed us to search the fMRI data not only for categorical effect of each type of uncertainty, but also for parametric effects of the level of each type of uncertainty (Levy et al., 2010; Bach et al., 2011b). For the rest of the paper the total number of Type 2 and Type 3 cards is referred to as the *level of ambiguity* or *level of conflict*.

Finally, the average winning probabilities of drawing Type 2 (and Type 3) cards from the deck in our design varied from 0.03 to 0.47; the average winning probabilities of Type 2 and Type 3 cards were matched across all lotteries. This is in contrast to most experimental designs that only examine ambiguity around an average winning probability of 0.5 (e.g., Ellsberg, 1961). A limited number of studies that did examine ambiguity attitudes around probabilities other than 0.5 found that participants do not necessarily demonstrate ambiguity aversion in their choices if average winning probabilities are lower than 0.35 (Kahn and Sarin, 1988; Maafi, 2011). Thus, we did not necessarily expect our participants to choose in an ambiguity avoidant manner in our experiment. No prior study has investigated similar probability ranges under conflict, and therefore whether our participants would exhibit conflict avoidance remained an open question.

Participants were told that they would not receive any feedback while in the scanner on whether they had won or lost the gamble. Instead, after completion of the fMRI session, four trials (one from each condition) were chosen randomly, and participants drew a card from each of the four decks if they chose to gamble, or received $3, if they chose the sure gain. For each draw predicted correctly during the fMRI session, they were paid $10; for each incorrect prediction, they were not paid. Overall, the participant payments ranged from $10 to $50 (mean payment = $38). Before the fMRI session, participants practiced on a computer, and were quizzed on how well they understood the instructions. Only after they had answered all of the questions correctly were they invited to perform the task in the scanner.

Each gamble appeared on the screen for 6500 ms, during which participants pressed one of four buttons to either choose the sure gain, or indicate their guess for the type of card that will be drawn when the lottery is played (Type 1, 2 or 3, see Figure 1). Each gamble presentation was followed by 1000 ms of fixation. Fixation-only trials of the same length as the gamble trials were also included. Gamble and fixation trials were pseudorandomly ordered according to a simulation algorithm to maximize estimation efficiency (AFNI scripts available at http://afni.nimh.nih.gov/afni/doc/howto/3); eight trials of each of the five trial types (risk, ambiguity, conflict, ignorance, and fixation) were presented in each run for a total of 8 min. Participants underwent four runs. Half of the participants saw the display with a sure gain option on the right, and another half saw the display with a sure gain option on the left.

### Imaging

A 3-tesla Siemens Trio MRI scanner with an eight-channel parallel head coil was used. For each run, 200 echo planar images were acquired (2500-ms TR, 30-ms TE, 81° flip angle, 38 axial slices, 64 × 64 matrix, 3.5-mm^3^ resolution). A T1-weighted MPRAGE anatomical scan with 1-mm^3^ voxels and a 1-min field-map scan were collected for each participant. Stimuli were presented using a high-resolution rear-projection system (Avotec, Stuart, FL) with button presses recorded using a four-button fiber-optics response pad. E-Prime software (Psychology Software Tools, Pittsburgh, PA) controlled the stimulus presentation and the recording of responses with each trial triggered by a scanner-generated optical pulse.

### Choice data analysis

#### Descriptive measures of choice behavior

First, we looked at how different types of uncertainty about the composition of cards of Type 2 and Type 3 affected the general choice patterns of our participants (for more details see Supplementary Materials S.2.a). Under risk, a risk-neutral decision maker would choose the option of the higher objective expected value, defined as the probability of a gain multiplied by the magnitude of that gain. In our task, such a decision-maker should choose to bet on cards of Type 2 and Type 3 ten times. Participants who chose risky lotteries less often are termed “risk averse,” and those who chose the lotteries more often are termed “risk-seeking.” Individual risk attitude (RA) thus was defined as

$$\begin{array}{l} RA=10-Number\;of\;bets\;on\;cards\;2\;or\;3\;under\;risk \end{array}$$

An ambiguity-neutral decision maker would make the same choices in ambiguous trials and in risky trials. To estimate ambiguity attitudes (AA) we therefore compared how often each participant bet on cards of Type 2 and Type 3 under ambiguity and under risk. We used the same approach to estimate individual conflict attitudes (CA).

$$\begin{array}{l} AA=Number\;of\;bets\;on\;cards\;2\;or\;3\;under\;risk\;\\ -Number\;of\;bets\;on\;cards\;2\;or\;3\;under\;ambiguity \end{array}$$

$$\begin{array}{l} CA=Number\;of\;bets\;on\;cards\;2\;or\;3\;under\;risk\;\\ -Number\;of\;bets\;on\;cards\;2\;or\;3\;under\;conflict\; \end{array}$$

Positive RA, AA, and CA values reflect uncertainty aversion, and negative values reflect uncertainty seeking (for more details see Supplementary Materials S.2.a).

#### Model-based analysis of choice behavior

The model-free descriptive approach relies on less restrictive assumptions than a model-based approach. These measures, however, do not account for non-linearities in individual sensitivity to rewards or for stochasticity in the choice behavior. We therefore also conducted a model-based analysis of the choice data that better accounts for these factors, as detailed in Supplementary Materials (S.2.b). In this additional analysis, we relied on a multinomial logit model (Congdon, 2003) that generalizes the model used in Hsu et al. (2005). We fitted the observed frequency of choice of each option (sure gain, Type 1 card, Type 2 card, and Type 3 card) with a logistic function of the relative utility of each option:

$$\begin{array}{l} p_{ij}=\exp\left(U_{ij}\right)∕\left(\sum_{k}\exp\left(U_{ik}\right)\right), \end{array}$$

where *U*_*ij*_ is the *i*th individual's subjective expected utility of the *j*th alternative. The expected utilities, in turn, are determined primarily by weights

$$\begin{array}{l} W_{ij}=x_{j}^{\theta_{i}}\pi_{j}^{\gamma_{ij}}, \end{array}$$

where *x*_*j*_ is the monetary payoff for the *j*th choice option (*j* = 1 for sure gain, *j* = 2 for Type 1, *j* = 3 for Type 2 or Type 3), π_*j*_ is the probability of winning that payoff, θ_*i*_ is subject *i*'s monetary utility parameter, and γ_*ij*_ is subject *i*'s probability weighting parameter for the jth task condition (*j* = 1 for ambiguity gambles, *j* = 2 for conflict gambles, and *j* = 3 for ignorance gambles; for all risk gambles γ_*i*_ = 1, to make the model identifiable).

Parameters of interest (γ_*A*_, ambiguity attitudes, and γ_*C*_, conflict attitudes) were estimated as individual utility functions, and can be interpreted as degrees of pessimism/optimism about the unknown probabilities under each type of uncertainty (further details are available in the Supplementary Materials S.2.b).

### fMRI data analysis

fMRI data were analyzed with the BrainVoyager QX 2.2 software package (Brain Innovation). Preprocessing of functional scans included discarding the first four volumes, slice scan time correction, three-dimensional motion correction, linear trend removal, high pass filtering of frequencies below three cycles per scan, and AR(1) removal. The images were then coregistered with each participant's high resolution anatomical scan, rotated into the anterior commissure–posterior commissure plane, and normalized into Talairach space (Talairach and Tournoux, 1988).

#### Statistical maps

Statistical analysis was based on a general linear model (Friston et al., 1995). The main model consisted of three groups of predictors: (1) to examine the main effect of each type of uncertainty, four dummy predictors for mean activation in risky trials (R), ambiguous trials (A), conflict trials (C), and ignorance trials (I); (2) to evaluate the effect of increasing level of each type of uncertainty, four parametric predictors for the total number of Type 2 and Type 3 cards (the level of uncertainty, see Section Procedure and Stimuli and Figure 1), for risky trials (R level), for ambiguity trials (A level), for conflict trials (C level), and for ignorance trials (I level); (3) to covary task difficulty across trial types, a regressor based upon an impulse model that occurs in all risk, ambiguity, ignorance, and conflict trials with a parametric value equal to the trial response time (RT). The conflict stimuli present more information on the screen than the other stimuli; thus, the participants may require more deliberation in conflict condition. This may lead to various confounds in fMRI analyses; including trial-by-trial RT as a regressor in GLMs during fMRI analyses may help to reduce these confounds (Yarkoni et al., 2009).

Each predictor time course was obtained by convolving boxcar time course with a double-gamma hemodynamic response function. Main effect boxcar predictors were defined by setting values to 1 at time points at which the modeled condition was defined and 0 at all other time points. Parametric predictors were mean-centered for each scan. Activation during inter-trial intervals and fixation period trials served as baseline. The activity time course of each voxel in each run was converted to percent signal change (PSC), and the main model was independently fitted to each voxel's PSC. The general linear model (GLM) analysis yielded regression coefficients (i.e., parameter estimates, PE) for each participant, that were used in a series of group-level random-effects analyses.

#### Region of interest analysis

In addition to traditional whole brain analyses, we employed a conservative *out-of-sample confirmation* approach to our region-of-interest (ROI) analysis. To this end we split all participants into two groups, matched on age, gender, and attitudes toward different types of uncertainty (Supplementary Materials, S.4). First (*exploratory analysis*), we performed a voxel-by-voxel whole-brain search on data from one group only to identify candidate ROIs. For each such region we created a sphere mask with a center at a region's peak and a radius of 10 voxels to be used during the confirmatory step. Second (*confirmatory analysis*), we sampled the time course from the second group of participants in each ROI, averaged it across all voxels, and fitted it with the main model. Only ROIs in which these confirmatory analyses yielded the same results as the exploratory analyses are reported below. We also report the total number of clusters revealed during the exploratory step. We chose the out-of-sample confirmation procedure as an alternative to the commonly accepted cluster size correction to address the multiple comparison problem (Forman et al., 1995) for the following reason. The unintended negative consequences of the focus on decreasing Type I error-rates is increased Type II error-rates (i.e., missing real effects), because for a fixed sample size Type I and Type II error-rates have a hydraulic trade off relationship. This tradeoff results in a bias toward studying large rather than small effects, a bias toward observing sensory and motor processes rather than complex cognitive and affective processes (a focus of this study), and deficient meta-analyses (Lieberman and Cunningham, 2009). Lieberman and Cunningham (2009) further recommend “placing a greater emphasis on replication and meta-analysis to determine which effects are real, and less emphasis on trying to determine the final truth from individual studies” (p. 427). Our approach to the data analyses follows their recommendations.

We primarily focus on two types of regions of interest (ROIs). First, to test whether a largely overlapping set of brain regions is involved in the processing of ambiguity and conflict we localized brain regions where activation was sensitive to A level and C level across trials on a group level.

Second, to test whether behavioral measures of attitudes toward ambiguity and conflict are predicted by activation in overlapping sets of regions we identified brain regions that predicted participant specific ambiguity/conflict attitudes. To that end we conducted a whole brain analysis, searching for voxels in which activation under ambiguity/conflict correlated with *model-based* measures of ambiguity/conflict attitudes (γ_*A*_ and γ_*C*_) across participants.

Calculation of significance values in the activation maps was based on the individual voxel significance and on the minimum cluster size (Forman et al., 1995). The probability of a false positive was determined from the frequency count of cluster sizes within the entire brain using a Monte Carlo simulation (using the Brain Voyager QX 2.2 software package).

#### Replication of the results of prior studies of decision making under ambiguity

A major concern in any neuroeconomics study is generalizability of the results across a variety of decision tasks. Therefore, even though they are not central to our research goal, we also conducted some of the fMRI analyses that have been employed in prior studies of decision making under ambiguity that have used decision tasks different from ours (we are not aware of any neuroeconomics study of decision making under conflict we can compare our results to).

First, we directly compared activation under ambiguity to activation under risk (referred to as the main effect of ambiguity). Such effects have been found in the posterior inferior frontal, posterior parietal cortex, dorsolateral prefrontal cortex, and the anterior insula (Hsu et al., 2005; Huettel et al., 2005, 2006; Rustichini et al., 2005; Bach et al., 2009; Levy et al., 2010). Note that Bach et al. (2011a) and (Lopez-Paniagua and Seger, 2013) demonstrate that activation revealed by the contrast of ambiguity vs. risk and activation that is sensitive to the level of ambiguity engage different brain areas. Second, we searched for brain regions where the main effect of ambiguity correlated with behavioral attitudes toward ambiguity (Huettel et al., 2006).

#### Potential confound: Correlation with subjective value

Finally, to rule out the possibility that some of the revealed effects would simply reflect changes in subjective value, we tested for correlations between activity in each ROI and the subjective value of the chosen option under ambiguity and under conflict. To that end, we constructed a second GLM that consisted of eight predictors: four dummy predictors for mean activation in risky trials, ambiguous trials (A), conflict trials (C), and ignorance trials, and four parametric parameters determining the subjective value of the chosen option under risk, under ambiguity, under ignorance, and under conflict. To construct the subjective value of each gamble for each individual, we used the same model that we employed to analyze the choice data (Supplementary Materials S.2.b), and model-based measures of individual attitudes toward different types of uncertainty that we derived from the individual choice data.

## Results

### Behavior

On each trial, participants chose between receiving a sure payoff and gambling on the type of card that they will draw from a mixed deck (Figure 1). The deck contained 100 cards of three types. The number of cards of one type was known (Type 1). However, the numbers of cards of the two other types (Types 2 and 3) were not precisely known: on *ambiguous* trials the combined number of cards of these two types was provided (Figure 1A), while on *conflict* trials conflicting numbers from two different sources were provided for each type (Figure 1B). Choice data were recorded and analyzed.

#### Response time by uncertain conditions

We compared response times under risk, ambiguity, and conflict, using a paired *t*-test. Response time under risk was significantly shorter than under ambiguity [*t*_(31)_ = −3.139, *p* < 0.01, Cohen's *d* = −0.555] or conflict [*t*_(31)_ = −4.714, *p* < 0.01, Cohen's *d* = −0.83]. Response time under ambiguity did not differ significantly from response time under conflict [*t*_(31)_ = −1.077, *p* = 0.29, Cohen's *d* = −0.19]. Response time under ambiguity did not significantly correlate with ambiguity level (*r* = −0.08, *p* = 0.66); response time under conflict did not significantly correlate with conflict level (*r* = 0.08, *p* = 0.65).

#### Descriptive measures of choice behavior

If risk, ambiguity, and conflict affect decision making in the same manner then general choice patterns under risk, ambiguity, and conflict should not differ significantly. A group level analysis of the choice data, however, revealed that on average participants bet on cards of Type 2 and Type 3 under conflict significantly less often than under risk, but under ambiguity significantly more often than under risk or conflict (mean for risk = 5.36 ± 0.62, mean for ambiguity = 8.58 ± 0.74, mean for conflict = 4.43 ± 0.67, out of 32 total choices, *p* < 0.01 for each contrast), which suggests that our participants chose differently under risk, ambiguity, and conflict. Next, we computed model-free estimates of individual risk attitudes (RA), ambiguity attitudes (AA), and conflict attitudes (CA), as detailed in Section Materials and Methods. RA, AA, or CA of 0 implies a “rational” agent, whose choices are not affected by uncertainty, while RA, AA, or CA < 0 implies tolerance to risk, ambiguity, or conflict, and RA, AA, or CA > 0 implies aversion to risk, ambiguity, or conflict. The CA of two participants and AA of one participant were classified as outliers (more than three standard deviations from the mean); these participants were removed from further analyses (see Supplementary Materials S.3.c). The remaining 30 participants, on average, were risk averse [mean RA = 4.57 ± 0.65, *t*_(29)_ = 7.029, *p* < 0.001, Cohen's *d* = 1.283] and conflict averse [mean CA = 1.00 ± 0.32, *t*_(29)_ = 3.084, *p* = 0.004, Cohen's *d* = 0.563] but ambiguity seeking [mean AA = −3.22±0.52, *t*_(29)_ = −6.198, *p* < 0.001, Cohen's *d* = −1.132], and individual risk, ambiguity, and conflict attitudes did not correlate with each other (Supplementary Materials, Table S.3.c.2, Figure S.3.c.5). This contradicts the implication of the hypothesis that risk, ambiguity and conflict affect decision making in the same manner; instead it suggests dissociation between these three processes.

#### Model-based analysis of choice behavior

Model-free measures of attitudes toward uncertainty do not account for non-linearities in individual sensitivity to rewards or for stochasticity in the choice behavior. We therefore conducted a model-based analysis of the choice data that better accounts for these factors, as detailed in Supplementary Materials (S.2.b). The main parameters of interest were model-based measures of attitudes toward ambiguity (γ_*A*_) and attitudes toward conflict (γ_*C*_); γ_*A*_(γ_*C*_) >1, = 1, < 1 implies ambiguity (conflict) aversion, neutrality, seeking.

**Figure 2:**
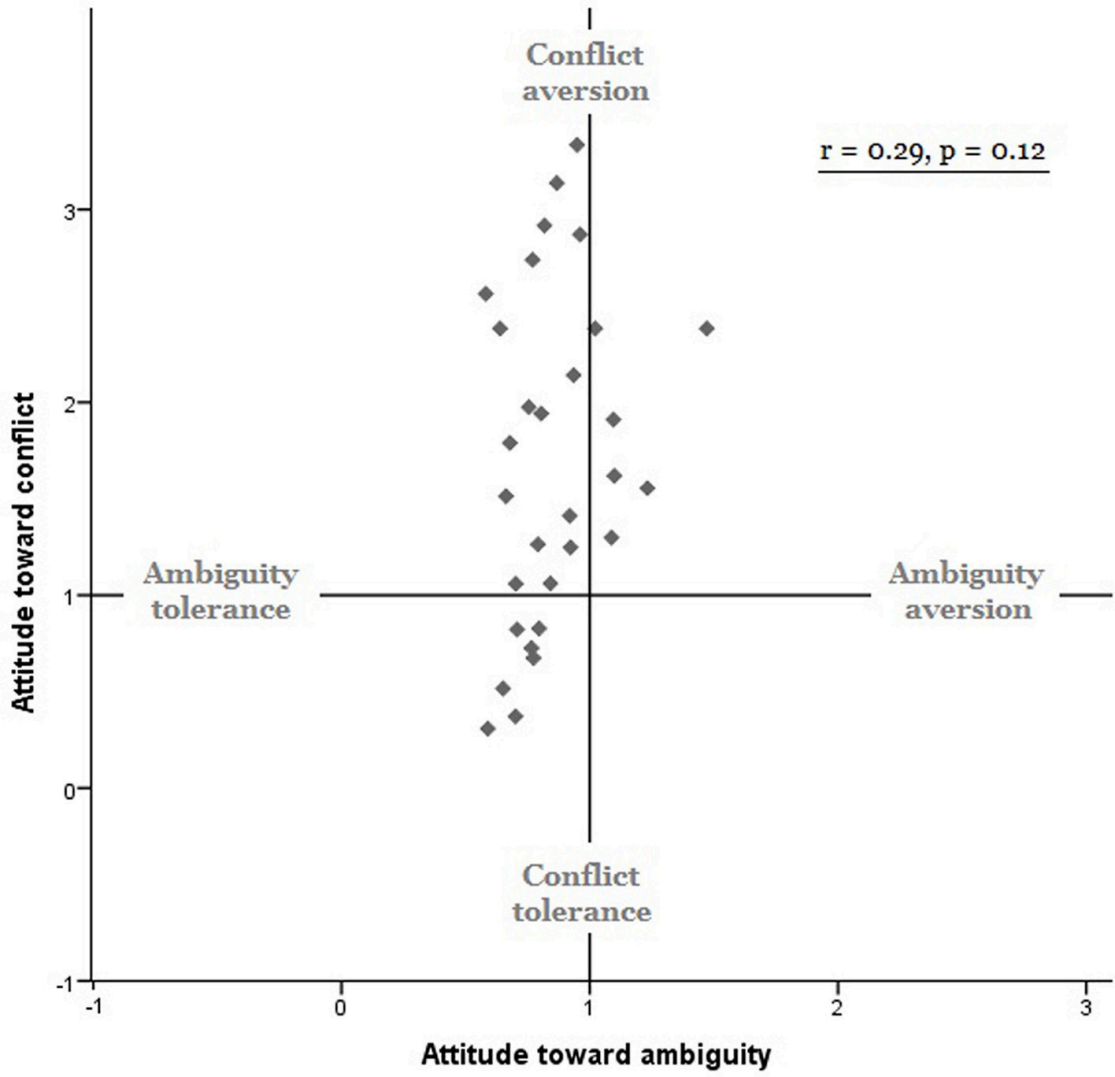
**Behavioral results**. Model-based measures of ambiguity attitudes (γ_*A*_, *x* axis) and conflict attitudes (γ_*C*_, *y* axis). Ambiguity attitudes did not correlate with conflict attitudes (*r* = 0.29, *p* = 0.12).

Results of the model-based analyses confirmed the results of the model-free analyses: (1) our participants on average were conflict averse [mean γ_*C*_ = 1.69 ± 0.16, *t*_(29)_ = 4.41, *p* < 0.001, Cohen's *d* = 0.805] but ambiguity seeking [mean γ_*A*_ = 0.85 ± 0.20, *t*_(29)_ = −4.01, *p* < 0.001, Cohen's *d* = −0.732]; and (2) individual attitudes toward ambiguity did not correlate with individual attitudes toward conflict [*r* = 0.29, *p* > 0.12, Figure 2]. Attitudes toward ambiguity were distributedlog-normally in our sample, therefore in all further analyses we used a log transformation. It is worth mentioning that including two previously excluded outliers in the whole brain analyses lead to the same, but more statistically significant, behavioral patterns.

The lack of ambiguity aversion in our data is in contrast to the results of the majority of prior studies (Camerer and Weber, 1992), and might appear somewhat surprising. There are two potential explanations for this finding. First, recall that a small number of studies that examined ambiguity attitudes around probabilities other than 0.5 found that participants do not necessarily demonstrate ambiguity aversion in their choices if average winning probabilities are lower than 0.35 (Kahn and Sarin, 1988; Maafi, 2011). In our design, the average winning probabilities of drawing Type 2 (and Type 3) cards from the deck in our design varied from 0.03 to 0.47. Consequently, we did not necessarily expect our participants to choose in an ambiguity avoidant manner. Interestingly, even under low average winning probabilities, we observe strong conflict avoidance. This suggests that manipulating the average winning probabilities affects ambiguity and conflict attitudes differently, which could be a topic for future research.

Second, the comparative ignorance hypothesis of ambiguity aversion (Fox and Tversky, 1995) suggests that ambiguity aversion is produced by a comparison with “less ambiguous events.” Our data, consistently with prior research on decision making under conflict (Smithson, 1999; Cabantous, 2007; Baillon and Cabantous, 2009; Cabantous et al., 2011), indicate that decision makers tend to avoid conflict more strongly than ambiguity. Possibly, conflict gambles in comparison to ambiguous gambles appeared to our participants so unattractive that this comparative context produced not only strong conflict aversion but also ambiguity seeking behavior.

Both hypotheses, as well as other potential explanations, cannot be tested with the available data and call for further research.

### fMRI

#### Neural correlates of uncertainty levels

To identify brain regions involved in the processing of ambiguous and conflicting information we employed a conservative out-of-sample confirmation approach (see Section Materials and Methods). Only ROIs that passed this strict criterion are reported below.

**Figure 3:**
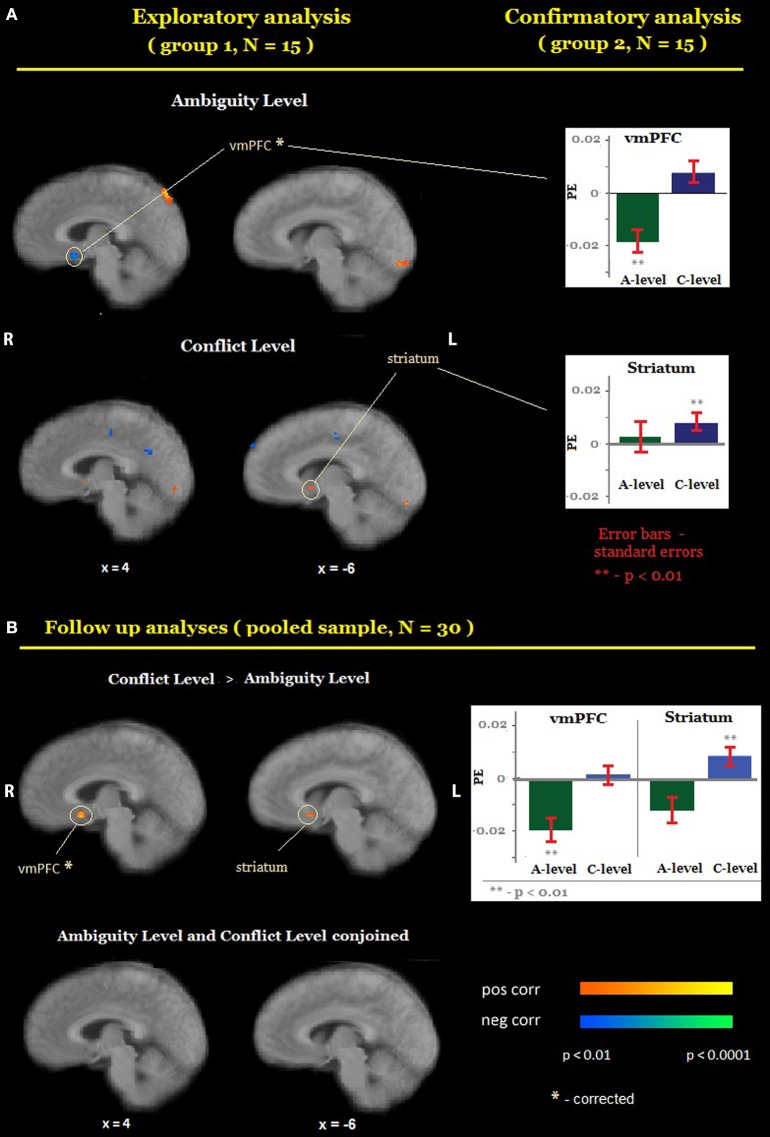
**Encoding of ambiguity and conflict**. **(A)** Random-effects group analysis showing areas that are correlated with the level of ambiguity (**A**, top) and conflict (**A**, bottom)*. First* (*exploratory analysis*), we analyzed data from one group only to identify candidate ROIs (A, left). For each such region we created a mask with a center at a region's peak and a radius 10 voxels. Second (*confirmatory analysis*), we sampled a time course from the second group of participants in each ROI, averaged it across all voxels, and fit it with the main model (A, right). Bars represent group average general linear model (GLM) coefficients of ambiguity and conflict levels in each ROI; the error bars represent standard errors. Only ROIs in which confirmatory analyses yielded the same results as exploratory analyses are reported in the figure. **(B)** Random-effects group analysis showing areas that are correlated with the level of conflict significantly more strongly than with the level of ambiguity (**B**, top), and areas that correlated similarly with both the level of ambiguity and the level of conflict (**B**, bottom). The functional maps are superimposed on a mean normalized anatomical image. vmPFC, ventromedial prefrontal cortex; L, left; R, right.

First, we localized areas whose activity was correlated with the level of ambiguity or the level of conflict (Figure 3). Exploratory analyses identified six candidate ROIs for A-level [located in the left dorsolateral prefrontal cortex, ventromedial prefrontal cortex (vmPFC), the left temporal cortex, the left and the right inferior parietal cortex, and the posterior parietal cortex] and 12 candidate ROIs for C-level [in the right middle frontal gyrus, the right dorsolateral prefrontal cortex, the left ventral striatum, the right temporal cortex, the posterior cingulate, the bilateral insula, the right parietal (BA 2), and the left parietal (BA 40)]. Confirmatory analyses revealed that (1) higher activation was associated with *lower* levels of ambiguity (Figure 3A, top) in the ventromedial prefrontal cortex (vmPFC, *x* = 4, *y* = 10, *z* = −10), and (2) higher activation was associated with higher levels of conflict in the left ventral striatum (*x* = −5, *y* = 7, *z* = −3). As a follow up analysis, we conducted a whole-brain search on the pooled sample (*N* = 30) for regions where correlation between activation and the level of conflict was significantly different from the correlation between activation and the level of ambiguity (C-level > A-level). This analysis confirmed that vmPFC and the left ventral striatum respond differently to the ambiguity and conflict levels (Figure 3B, top). Finally, we conducted a whole-brain search on the pooled sample for regions where activation correlated similarly with both ambiguity and conflict level (conjoined C-level and A-level). This analysis did not reveal any significant correlation in either the vmPFC or the striatum (Figure 3B, bottom) or in any other brain region.

While the statistical maps revealed non-overlapping areas for ambiguity and conflict levels, it is still possible that the average activation in these areas that seem to be unique for one type of uncertainty also provides information about the other type. We therefore sampled activation in each of the ROIs whose activity was correlated with the level of one type of uncertainty, and examined its correlation with the level of the other type of uncertainty. Activation in vmPFC did not correlate significantly with the level of conflict and activation in the left ventral striatum did not correlate significantly with the level of ambiguity (Table 1, Figure 3).

**Table 1:** **Correlations between neural activation and levels of uncertainty and behavioral parameters**.

**Region**	**Talairach coordinates**			**Size**	**Correlation with the level of uncertainty**		**Correlation with the attitudes toward uncertainty**	
	***x***	***y***	***z***	**[mm^3^]**	**PE ambiguity level**	**PE conflict level**	**PE ambiguity with ambiguity aversion (ln γ_1_)**	**PE conflict with conflict aversion (γ_2_)**
vmPFC and ACC	4	10	−10	254	−**0.015**	*0.001*	*0.14*	*−0.22*
Medial prefrontal cortex	4	56	17	463	−0.004	0.003	**0.61**	*−0.05*
Left ventral striatum	−5	7	−3	148	*−0.006*	**0.002**	*0.086*	*0.098*
Right ventral striatum	7	12	5	247	*−0.004*	*−0.002*	*0.018*	−**0.67**

*vmPFC, ventromedial prefrontal cortex; ACC, anterior cingulate cortex; in bold—statistically significant correlations (p < 0.0005), in italic—statistically non-significant correlations (p > 0.15), random effects analysis*.

These results contradict the hypothesis that a largely overlapping set of brain regions is involved in the processing of ambiguity and conflict, and suggest that vmPFC and striatum play a unique role in the processing of either ambiguity or conflict.

#### Neural correlates of individual uncertainty attitudes

While correlation with the level of ambiguity or conflict is a prerequisite for defining an area as involved in the processing of that attribute, we also expect activity in such an area to be correlated with behavioral attitudes across participants. We therefore sampled activity from our two ROIs (vmPFC and striatum) and examined their correlation with ambiguity and conflict attitudes across our participants. None of these ROIs correlated with ambiguity or conflict attitudes across participants (Table 1).

**Figure 4:**
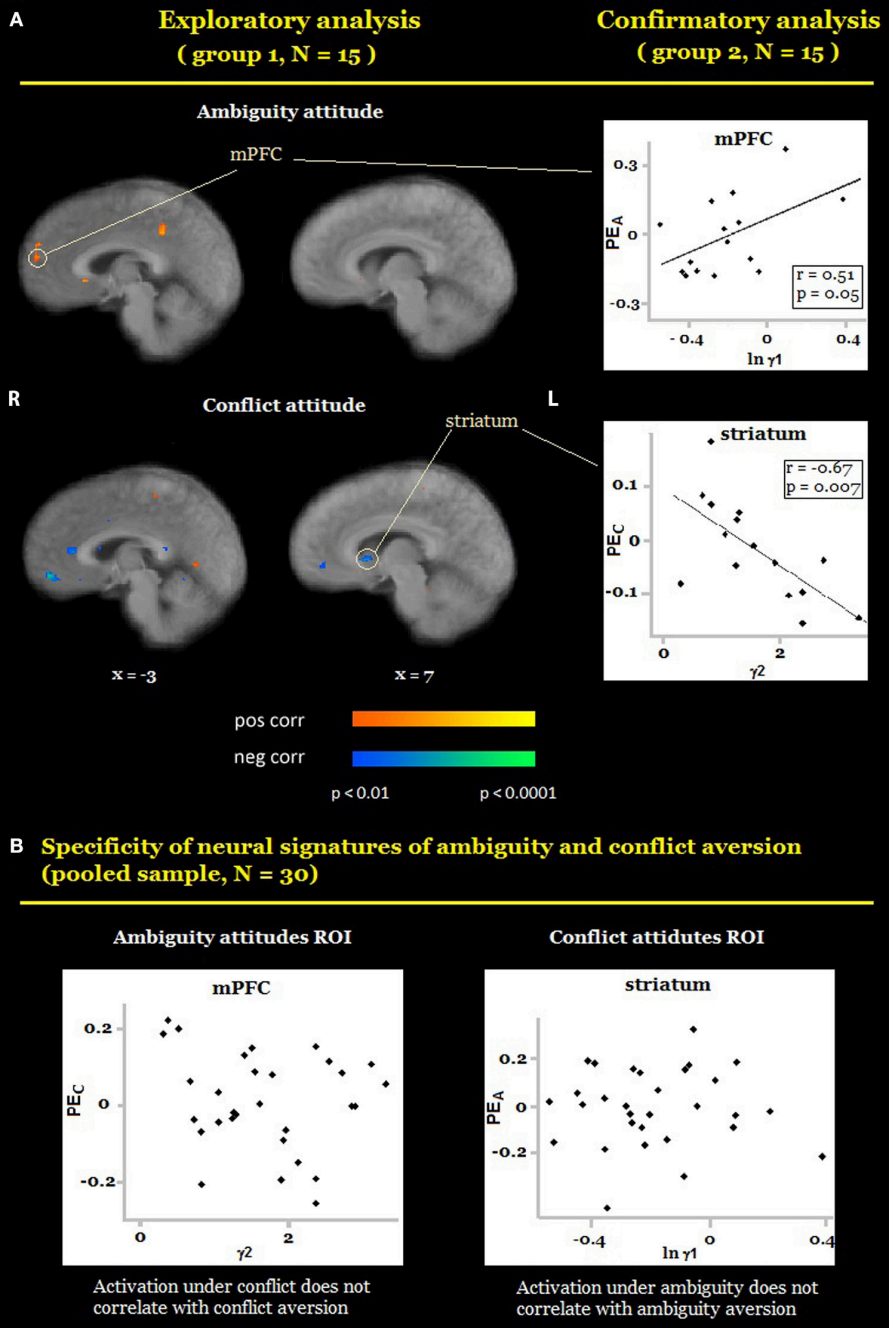
**Preferences for ambiguity and conflict**. **(A)** Random-effects group analysis showing areas that are correlated with ambiguity attitudes (**A**, top) and conflict attitudes (**A**, bottom)*. First* (*exploratory analysis*), we analyzed data from one group only to identify candidate ROIs (**A**, left). For each such region we created a mask with a center at a region's peak and a radius 10 voxels. Second (*confirmatory analysis*), we sampled a time course from the second group of participants in each ROI, averaged it across all voxels, and fit it with the main model (**A**, right). Scatterplots represent relation between participants' general linear model (GLM) coefficients of main effects of ambiguity and conflict in each ROI and participants' ambiguity and conflict attitudes. Only ROIs in which confirmatory analyses yielded the same results as exploratory analyses are reported in the figure. Activation under ambiguity correlated with ambiguity aversion in medial prefrontal cortex (MPFC); activation under conflict correlated with conflict aversion in the ventral striatum. **(B)** Scatter plots depict the results of the test for specificity of neural signatures of behavioral measures. Participants' coefficients of main effects of conflict did not correlate with participants' conflict aversion in MPFC (**B**, left); participants' coefficients of main effects of ambiguity did not correlate with participants' ambiguity aversion in the ventral striatum (**B**, right). The functional maps are superimposed on a mean normalized anatomical image. L, left; R, right.

We also conducted a whole-brain analysis, searching for voxels in which activation under ambiguity/conflict was correlated with ambiguity/conflict attitudes (γ_*A*_/γ_*C*_) across participants using the out-of-sample confirmation approach (see Section Materials and Methods). Exploratory analyses revealed eight candidate ROIs for ambiguity attitudes (located in the medial prefrontal cortex, the superior frontal gyrus, the bilateral precentral cortex (BA 6), the bilateral temporal cortex, the right parietal cortex (BA 7), the right posterior parietal cortex), and nine candidate ROIs for conflict attitudes (the right ventral striatum, the bilateral middle frontal gyrus (BA 8 and BA 9), the bilateral precentral cortex (BA 6), the bilateral amygdala, the right temporal cortex). The confirmatory analyses revealed that ambiguity and conflict attitudes predicted activation in two different regions (Table 1, Figure 4A). Ambiguity aversion correlated positively with activation under ambiguity in the medial prefrontal cortex (mPFC, Figure 4A top), activation under conflict in this ROI did not correlate with conflict attitudes (Figure 4B, left). Conflict aversion correlated negatively with activation under conflict in the right ventral striatum (Figure 4A, bottom), but activation under ambiguity in the striatum did not correlate with ambiguity attitudes (Figure 4B, right). As a follow-up analysis, we conducted a whole-brain search on the pooled sample (*N* = 30) for regions where activation under ambiguity/conflict was correlated with ambiguity/conflict attitudes (γ_*A*_/γ_*C*_) across participants. These analyses confirmed that mPFC and ventral striatum correlate differently with ambiguity and conflict attitudes across participants. Finally, in each ROI identified as sensitive to ambiguity, we examined whether its activation also correlated with the level of conflict, and vice versa. None of these correlations was significant (Table 1). These results contradict the hypothesis that behavioral attitudes toward ambiguity and conflict should be predicted by the activation in a largely overlapping set of regions, and suggest that mPFC and striatum play a unique role in the processing of either ambiguity or conflict.

Including two previously excluded outliers in the whole brain analyses lead to the same, but more statistically significant, activation patterns.

#### Replication of the results of prior studies of decision making under ambiguity

Our follow up analyses replicated the increased activation during decisions involving ambiguity compared to those involving risk in the bilateral posterior parietal cortex, left amygdala, and the posterior inferior frontal sulcus (Supplementary Materials, figure S.5.a, Hsu et al., 2005; Rustichini et al., 2005; Huettel, 2006; Huettel et al., 2006; Bach et al., 2009, etc.). Our analyses also replicated the positive correlation between the neural ambiguity effect (compared to risk) and ambiguity aversion in the right posterior inferior frontal sulcus (Supplementary Materials, Figure S.5.b, Huettel et al., 2006). The last result is particularly important in the context of the observed lack of ambiguity aversion in our experiment. It suggests that the neural signatures of ambiguity attitude are not only robust across experimental designs, but also across behavioral effects (aversion vs. seeking).

#### Potential confound: Correlation with subjective value

The vmPFC and striatum have been linked to the encoding of subjective value (Kable and Glimcher, 2009; Levy et al., 2010; Bartra et al., 2013). Arguably, both the level of ambiguity/conflict and individual ambiguity/conflict attitudes are likely to correlate strongly with the subjective value of the gambles, the observed sensitivity to ambiguity/conflict level and ambiguity/conflict attitudes might simply reflect sensitivity to changes in subjective value. To examine this possibility, we looked for correlation between the activity in each ROI and the subjective value of the chosen option under ambiguity and under conflict. To construct the subjective value of each gamble for each individual, we used the same model that we employed to analyze the choice data (Supplementary Materials S.2.b), and model-based measures of individual attitudes toward different types of uncertainty that we derived from the individual choice data. Activation in none of these regions correlated with subjective value under either ambiguity or conflict.

## Discussion

### Summary of findings

**Figure 5:**
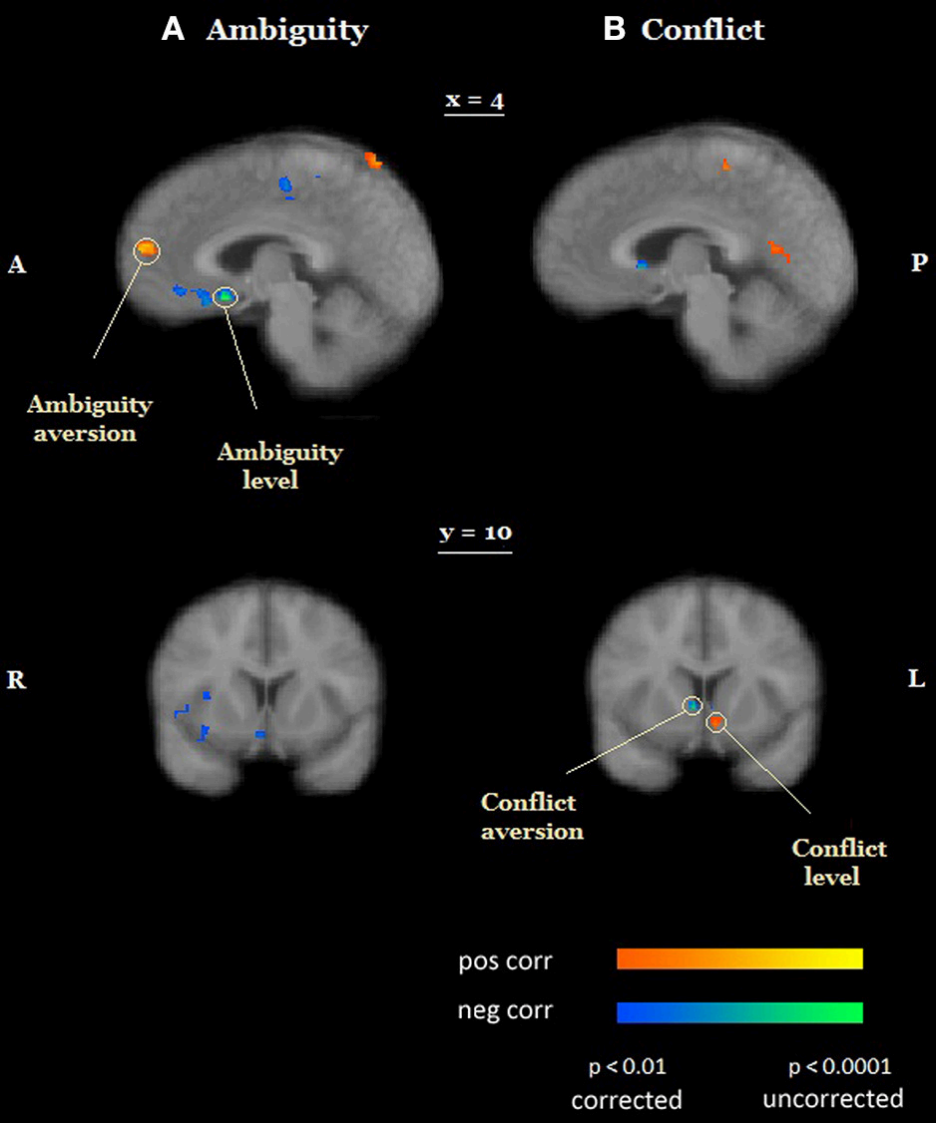
**Objective and subjective effects of ambiguity and conflict**. **(A)** Activation in neighboring regions of the medial prefrontal cortex was sensitive to both the level of ambiguity (ventromedial prefrontal cortex, vmPFC) and ambiguity attitudes (medial prefrontal cortex MPFC). **(B)** Activation in neighboring regions of the ventral striatum was sensitive to both the level of conflict (a more ventral region) and conflict attitudes (a more dorsal region). The functional maps are superimposed on a mean normalized anatomical image. L, left; R, right; A, anterior; P, posterior.

Most research on decision making under uncertainty assumes that conflicting information affects decision makers in the same way as ambiguous information does (Viscusi and Magat, 1992; Cameron, 2005). Our results suggest that this is not the case. Behaviorally, (1) participants avoided conflict more than ambiguity, even though ambiguity and conflict were matched on expected value and outcome variance under ambiguity was higher than outcome variance under conflict, and (2) ambiguity attitudes and conflict attitudes (by either model-free or model-based measures) were not correlated across participants. Neurally, activation in neighboring regions of the medial prefrontal cortex (Figure 5) was sensitive to both the level of ambiguity and ambiguity attitudes. Conversely, activation in neighboring regions of the striatum (Figure 5) was sensitive to both the level of conflict and conflict attitudes. Thus, our results are not compatible with the hypothesis that decision makers respond in the same manner to ambiguous and conflicting information, and suggest that attitudes toward conflict and ambiguity have distinct neurobiological signatures.

### Previous studies

While to the best of our knowledge this is the first fMRI study of decision making under conflict, it is just one of several studies of decision making under ambiguity. Our findings with regards to ambiguity replicate the most robust results from this literature. Similarly to Rustichini et al. (2005), Huettel et al. (2006), and Bach et al. (2009) we observe increased activation under ambiguity compared to risk in the bilateral posterior parietal cortex, and the right posterior inferior frontal cortex. Similarly to Huettel et al. (2006) we also find that the ambiguity effect (compared to risk) in the right posterior inferior frontal sulcus correlates positively with behavioral measures of ambiguity aversion. Our results are also compatible with the results of a recent study (Lopez-Paniagua and Seger, 2013) that finds a negative association between ambiguity levels and activation in the OFC region (*x* = −2, *y* = 36, *z* = −10) that is neighboring to the vmPFC region identified by our study (*x* = 4, *y* = 10, *z* = −10).

### Ambiguity but not conflict effect in PFC

Our results implicate two distinct regions (one in more anterior part of mPFC and one in vmPFC) of the prefrontal cortex (PFC) in ambiguity processing. Previous studies indicate a special role of various regions of PFC in ambiguity processing and suggest several potential explanations. First, a “task complexity hypothesis,” follows from the observation that PFC is organized in hierarchical manner in which different regions support various aspects of cognitive control (Koechlin and Summerfield, 2007; Badre and D'Esposito, 2009). As task demands increase, regions of the prefrontal cortex can be recruited in the posterior to anterior fashion. This “complexity hypothesis” is consistent with the positive correlation in mPFC between activation under ambiguity and individual ambiguity aversion observed in our study. Arguably, decision making under ambiguity may appear more complex for ambiguity-averse compared to ambiguity seeking participants, and therefore may require more effort and more cognitive control for more averse participants.

Second, a “valuation hypothesis” follows from ample evidence that suggests that the vmPFC (as well as the striatum) is a part of a general valuation system (Mohr et al., 2010; Bartra et al., 2013; Clithero and Rangel, 2013). Activity in these areas has been linked to various measures of stimulus value across a wide range of decision tasks, reward modalities and stages of decision making (Clithero and Rangel, 2013), including the expectation (Tom et al., 2007) of reward. It is modulated by various factors that affect value, including objective aspects, such as the magnitude and probability of the reward (Bartra et al., 2013), and subjective aspects, such as the individual weighting of delay to reward (Kable and Glimcher, 2007) or uncertainty in obtaining the reward (Levy et al., 2010), as well as behavioral preferences for non-monetary rewards (O'Doherty et al., 2002). To test the valuation hypothesis, we looked at correlations between the activity in the vmPFC (and the striatum) and the subjective value of the chosen option under ambiguity and under conflict. Activation in none of these regions correlated with subjective value under either ambiguity or conflict. Importantly, however, the monetary rewards in our task were not very high (the maximal winning was $10), and the lack of correlation might be simply due to low variability in the subjective value. Furthermore, in our decision task three options are associated with changing probabilities, and thus with changing subjective values. This is in contrast to the majority of prior studies that vary only one value-related parameter at a time (Levy et al., 2010). Encoding of values of multiple options may potentially lead to decreased specificity of the signal, which also can explain the lack of observed correlations between the activity in the vmPFC (and the striatum) and the subjective value of the chosen option under ambiguity and under conflict.

Third, Lopez-Paniagua and Seger (2013) suggested that ambiguity, and particularly partial ambiguity, may be perceived by participants as a situation associated with potentially knowable information. That is, under ambiguity, activation in PFC might reflect an active search for contextual cues that might reduce the level of ambiguity (Huettel et al., 2006). Our results do not necessarily contradict this hypothesis. Under both conflict and ambiguity, one is required to act based on some belief regarding the actual reward probability. Under ambiguity, however, this belief can be consistent with the imprecise information participants receive, if it belongs to the set of possible probabilities. Under conflict, on the other hand, any belief is at odds with at least one of the sources providing the information. Using the example from the introduction, if your friends agree that the two horses that you consider have performed equally inconsistently lately, you might form an expectation, although imprecise, about the winning odds of each of these horses, and make a decision about which horse to pick. In the case of conflict, however, you can either accept the advice from one of your friends, knowing that maybe the other one was right, or completely dismiss both. It is possible that these unavoidable difficulties in forming predictions under conflict are reflected in the differential activation patterns that we observed.

Finally, our findings are consistent with the results of a recent study by Lebreton et al. (2015), which linked activity in vmPFC to subjectively reported confidence in subjective valuation. Lower levels of ambiguity are likely to be associated with higher confidence in the subjective value of ambiguous options, while varying levels of conflict are not likely to affect subjective confidence in the value of conflicting options. Thus, the link between vmPFC activity and ambiguity, but not conflict, level, may reflect varying degree of confidence associated with varying level of ambiguity. Future studies need to test more accurately this intriguing possibility.

### Questions for follow up studies

To the best of our knowledge, this is the first study that compares effects of ambiguous and conflicting information about outcome probabilities on decision making, both behaviorally and neurally; additional follow up studies have to replicate our results before they may be deemed verified. Our results suggest several questions for such studies. First, there are several similarities and differences between informational conflict (Smithson, 1999; Cabantous, 2007; Cabantous et al., 2011) and cognitive conflict (Pochon et al., 2008; Marco-Pallarés et al., 2010). On the one hand, both conditions are associated with conflicting evidence from multiple sources. On the other hand, cognitive conflict may be potentially resolved with additional cognitive effort (e.g., attentional control or error monitoring, Petersen and Posner, 2012). Behaviorally, additional cognitive effort is reflected in longer response time under higher levels of cognitive conflict (Fan et al., 2002). Neurally, higher cognitive effort and error monitoring is reflected in selective involvement of anterior cingulate cortex (Swick and Turken, 2002). In contrast, informational conflict cannot be resolved with additional cognitive effort; only additional information from or about the conflicting sources may, potentially, be helpful. Thus, additional cognitive effort under these circumstances is not appropriate. It is therefore not surprising that our results do not reveal significant correlations between (1) the level of conflict and response time, and (2) the level of conflict and activation in the anterior cingulate cortex. However, our study was designed to contrast ambiguity and conflict, not to contrast informational and cognitive conflict. Future studies may carefully compare and contrast these two decision environments. For instance, it would be interesting to look at whether individual performance on the Stroop or Flanker tasks correlates with individual conflict attitudes. It would also be interesting to investigate a potential relationship between conflict aversion and effort avoidance, since both appear to relate to activation in similar regions of ventral striatum (Prévost et al., 2010).

Second, the magnitudes of potential outcomes in our experiment did not vary sufficiently, which limited our ability to test the interaction between value and uncertainty encoding in the vMPFC and the striatum, a question that is left for a follow up study.

Third, we did not inform study participants that all conditions were matched on expected value, and that outcome variance under ambiguity was higher than under conflict. One potential explanation of ambiguity and conflict aversion is an irrationally pessimistic belief that unknown odds are always not in one's favor. We chose to allow our participants to form their own beliefs about the underlying distribution under each type of uncertainty, so we could evaluate the effect of each type of uncertainty on these beliefs. It is unclear whether or not explicit knowledge about expected value and outcome variance across different conditions would affect individual behavioral patterns. A follow up study may research this.

Finally, in our task participants acted on average in an ambiguity seeking manner. We speculate that one potential explanation for this is a comparative ignorance hypothesis. Possibly, presenting both ambiguous and conflicting gambles to participants both suppressed ambiguity aversion and inflated conflict aversion. A follow up study may present ambiguous and conflict gambles in separate sessions, which may reduce the comparative effects and may lead to the more commonly observed ambiguity and conflict aversion patterns.

### Implications for clinical and basic research

The results of this study may offer insights into behavioral and neurobiological mechanisms underlying anxiety spectrum disorders. Intolerance of uncertainty has been repeatedly associated with such disorders (Tolin et al., 2003), but the present results suggest that it may be also important to investigate individual sensitivity to conflicting information and its relationship to anxiety-driven symptomatology. Indeed, an early study (Bugental et al., 1971) provided some evidence of a causal role of prolonged exposure to conflict in generating anxiety-driven aggressiveness. The same idea was extensively discussed in the context of “double bind” (Bateson et al., 1956)—an emotionally distressing communication dilemma, in which an individual receives two or more conflicting messages, in which one message negates the other. Double bind effectively creates a situation in which a successful response to one message results in a failed response to the other message, such that the individual will inevitably be wrong, regardless of response, similar to the conflict condition in our design. Bateson and colleagues (Bateson et al., 1956; Bateson, 1972) further hypothesized that prolonged exposure to double bind might be linked to schizophrenia and psychosis, although these hypothetical causal relations have never been directly tested.

Interestingly, a more recent study (Smithson, 1999) found that decision-makers tend to distrust the sources of conflicting messages much more than the sources of ambiguous but agreeing messages. This distinction between ambiguity and conflict may also be informative for assessing neurocognitive endophenotypes of clinical populations within the framework of computational neuropsychiatry (Montague et al., 2012). Finally, the distinction is also important for neuroeconomics and behavioral economic analyses. Applying models that focus exclusively on imprecise information to behavioral and cognitive processes under conflict is likely to lead to biased predictions and to model misspecification. Indeed, a case can be made for constructing normative decision making models that incorporate conflict along with ambiguity, and an initial attempt toward this goal has been described (Gajdos and Vergnaud, 2013; Smithson, 2013).

Our ability to make effective decisions is affected by the quality of information we receive. Understanding more clearly what these effects are will help us make better decisions despite the quality of information.

### Conflict of interest statement

The authors declare that the research was conducted in the absence of any commercial or financial relationships that could be construed as a potential conflict of interest.

## Abbreviations

A: ambiguity condition
AA: ambiguity attitude
A-level: level of ambiguity
ACC: anterior cingulate cortex
AFNI: Analysis of Functional NeuroImages
AR(1): an autoregressive process when only the previous term in the process and the noise term contribute to the output
BOLD: Blood-oxygenated-level dependent signal
C: conflict condition
CA: conflict attitude
C level: conflict level
fMRI: functional magnetic resonance imaging
GLM: generalized linear model
laINS: left anterior insula
lPAR: left parietal cortex
STG: superior temporal gyrus
lSTG: left superior temporal gyrus
MPRAGE: magnetization-prepared 180 degrees radio-frequency pulses and rapid gradient-echo sampling
MRI: magnetic resonance imaging
OFC: orbito frontal cortex
rlOFC: right lateral orbitofrontal cortex
PCC: posterior cingular cortex
PE: parameter estimate
PFC: posterior frontal cortex
Pifs: posterior inferior frontal sulcus
PSC: percent signal change
ROI: region of interest
SEU: Subjective Expected Utility Theory
STD: standard error
TE: Echo Time
TR: repetition time.
