# Supplementary material for: Neural Correlates of Decision-Making Under Ambiguity and Conflict
Source: Front Behav Neurosci. 2015 Nov 27;9:325. doi: 10.3389/fnbeh.2015.00325 (PMC4661279; doi:10.3389/fnbeh.2015.00325)
Supplement: Supplementary file 1 [file DataSheet1.DOCX]

**Supplementary Material**

**for**

**Neural correlates of decision-making under ambiguity and conflict**

**S.1. Full list of risky, ambiguous and conflict gambles used in the experiment.** Each series consists of 30 unique gambles. During the experiment, 32 gambles (8 gambles x 4 runs) were used from each condition. Therefore, 2 randomly selected gambles from each condition were used twice.

*Table S.1a. Full list of risky gambles.*

| **Card 1** | **Card 2** | **Card 3** | **Card 2 + Card 3** | **Total** |
| --- | --- | --- | --- | --- |
| 6 | 47 | 47 | 94 | 100 |
| 10 | 45 | 45 | 90 | 100 |
| 14 | 43 | 43 | 86 | 100 |
| 16 | 42 | 42 | 84 | 100 |
| 18 | 41 | 41 | 82 | 100 |
| 20 | 40 | 40 | 80 | 100 |
| 24 | 38 | 38 | 76 | 100 |
| 26 | 37 | 37 | 74 | 100 |
| 30 | 35 | 35 | 70 | 100 |
| 32 | 34 | 34 | 68 | 100 |
| 36 | 32 | 32 | 64 | 100 |
| 38 | 31 | 31 | 62 | 100 |
| 42 | 29 | 29 | 58 | 100 |
| 44 | 28 | 28 | 56 | 100 |
| 48 | 26 | 26 | 52 | 100 |
| 50 | 25 | 25 | 50 | 100 |
| 54 | 23 | 23 | 46 | 100 |
| 58 | 21 | 21 | 42 | 100 |
| 60 | 20 | 20 | 40 | 100 |
| 62 | 19 | 19 | 38 | 100 |
| 64 | 18 | 18 | 36 | 100 |
| 68 | 16 | 16 | 32 | 100 |
| 72 | 14 | 14 | 28 | 100 |
| 74 | 13 | 13 | 26 | 100 |
| 76 | 12 | 12 | 24 | 100 |
| 80 | 10 | 10 | 20 | 100 |
| 84 | 8 | 8 | 16 | 100 |
| 88 | 6 | 6 | 12 | 100 |
| 90 | 5 | 5 | 10 | 100 |
| 92 | 4 | 4 | 8 | 100 |
| **49.2** | **25.4** | **25.4** | **50.8** |  |

*Table S.1b. Full list of ambiguous gambles.*

| **Card 1** | **Card 2 + Card 3** | **Total** |
| --- | --- | --- |
| 7 | 93 | 100 |
| 9 | 91 | 100 |
| 14 | 86 | 100 |
| 16 | 84 | 100 |
| 18 | 82 | 100 |
| 21 | 79 | 100 |
| 23 | 77 | 100 |
| 26 | 74 | 100 |
| 30 | 70 | 100 |
| 32 | 68 | 100 |
| 35 | 65 | 100 |
| 39 | 61 | 100 |
| 42 | 58 | 100 |
| 44 | 56 | 100 |
| 48 | 52 | 100 |
| 51 | 49 | 100 |
| 53 | 47 | 100 |
| 58 | 42 | 100 |
| 60 | 40 | 100 |
| 62 | 38 | 100 |
| 64 | 36 | 100 |
| 67 | 33 | 100 |
| 72 | 28 | 100 |
| 75 | 25 | 100 |
| 76 | 24 | 100 |
| 80 | 20 | 100 |
| 83 | 17 | 100 |
| 88 | 12 | 100 |
| 90 | 10 | 100 |
| 93 | 7 | 100 |
| **49.2** | **50.8** |  |

*Table S.1c. Full list of conflict gambles*

| **Card 1 (A)** | **Card 2 (A)** | **Card 3 (A)** | **Card 1 (B)** | **Card 2 (B)** | **Card 3 (B)** | **Card 2 + Card 3** | **Total** |
| --- | --- | --- | --- | --- | --- | --- | --- |
| 4 | 59 | 37 | 4 | 37 | 59 | 96 | 100 |
| 10 | 56 | 34 | 10 | 34 | 56 | 90 | 100 |
| 15 | 54 | 31 | 15 | 31 | 54 | 85 | 100 |
| 16 | 51 | 33 | 16 | 33 | 51 | 84 | 100 |
| 18 | 50 | 32 | 18 | 32 | 50 | 82 | 100 |
| 22 | 48 | 30 | 22 | 30 | 48 | 78 | 100 |
| 25 | 47 | 28 | 25 | 28 | 47 | 75 | 100 |
| 26 | 46 | 28 | 26 | 28 | 46 | 74 | 100 |
| 29 | 44 | 27 | 29 | 27 | 44 | 71 | 100 |
| 30 | 43 | 27 | 30 | 27 | 43 | 70 | 100 |
| 36 | 40 | 24 | 36 | 24 | 40 | 64 | 100 |
| 38 | 38 | 24 | 38 | 24 | 38 | 62 | 100 |
| 42 | 36 | 22 | 42 | 22 | 36 | 58 | 100 |
| 44 | 34 | 22 | 44 | 22 | 34 | 56 | 100 |
| 46 | 33 | 21 | 46 | 21 | 33 | 54 | 100 |
| 52 | 30 | 18 | 52 | 18 | 30 | 48 | 100 |
| 54 | 28 | 18 | 54 | 18 | 28 | 46 | 100 |
| 58 | 26 | 16 | 58 | 16 | 26 | 42 | 100 |
| 61 | 25 | 14 | 61 | 14 | 25 | 39 | 100 |
| 62 | 23 | 15 | 62 | 15 | 23 | 38 | 100 |
| 63 | 20 | 17 | 63 | 17 | 20 | 37 | 100 |
| 68 | 20 | 12 | 68 | 12 | 20 | 32 | 100 |
| 72 | 17 | 11 | 72 | 11 | 17 | 28 | 100 |
| 76 | 15 | 9 | 76 | 9 | 15 | 24 | 100 |
| 77 | 15 | 8 | 77 | 8 | 15 | 23 | 100 |
| 78 | 13 | 9 | 78 | 9 | 13 | 22 | 100 |
| 84 | 10 | 6 | 84 | 6 | 10 | 16 | 100 |
| 86 | 8 | 6 | 86 | 6 | 8 | 14 | 100 |
| 90 | 6 | 4 | 90 | 4 | 6 | 10 | 100 |
| 94 | 4 | 2 | 94 | 2 | 4 | 6 | 100 |
| **49.2** | **31.3** | **19.5** | **49.2** | **19.5** | **31.3** | **50.8** |  |

*Table S.1.d. Split of ambiguity and conflict gambles by runs.*

|  | **Block** | | **Card 1** | **Card 1 +**  **Card 2** | **EV** | **Var.** | **Centered level of ambiguity** |  | **Block** | **Card 1** | **Card 2** | | **Card 3** | | **Card 1 +**  **Card 2** | **EV** | **Var.** | **Centered level of conflict** |
| --- | --- | --- | --- | --- | --- | --- | --- | --- | --- | --- | --- | --- | --- | --- | --- | --- | --- | --- |
|  |  |  |  |  |  |  |  |  |  |  | **A** | **B** | **A** | **B** |  |  |  |  |
| Scan 1 | | |  |  |  |  |  |  |  |  |  |  |  |  |  |  |  |  |
|  | | 17 | 90 | 10 | 0.5 | 0.05 | -47.63 |  | 3 | 52 | 30 | 18 | 18 | 30 | 48 | 2.4 | 0.36 | -12.38 |
|  | | 19 | 51 | 49 | 2.45 | 1.84 | -8.63 |  | 8 | 61 | 25 | 14 | 14 | 25 | 39 | 1.95 | 0.30 | -21.38 |
|  | | 20 | 67 | 33 | 1.65 | 0.80 | -24.63 |  | 15 | 68 | 20 | 12 | 12 | 20 | 32 | 1.6 | 0.16 | -28.38 |
|  | | 21 | 32 | 68 | 3.4 | 3.63 | 10.38 |  | 16 | 42 | 36 | 22 | 22 | 36 | 58 | 2.9 | 0.49 | -2.38 |
|  | | 33 | 18 | 82 | 4.1 | 5.33 | 24.38 |  | 45 | 22 | 48 | 30 | 30 | 48 | 78 | 3.9 | 0.81 | 17.63 |
|  | | 37 | 7 | 93 | 4.65 | 6.90 | 35.38 |  | 47 | 58 | 26 | 16 | 16 | 26 | 42 | 2.1 | 0.25 | -18.38 |
|  | | 38 | 53 | 47 | 2.35 | 1.69 | -10.63 |  | 63 | 10 | 56 | 34 | 34 | 56 | 90 | 4.5 | 1.21 | 29.63 |
|  | | 58 | 21 | 79 | 3.95 | 4.94 | 21.38 |  | 64 | 4 | 59 | 37 | 37 | 59 | 96 | 4.8 | 1.21 | 35.63 |
| **Mean** | | | **42.38** | **57.63** | **2.88** | **3.15** | **0.00** |  |  | **39.63** | **37.50** | **22.88** | **22.88** | **37.50** | **60.38** | **3.02** | **0.60** | **0.00** |
|  | |  |  |  |  |  |  |  |  |  |  |  |  |  |  |  |  |  |
| Scan 2 | | |  |  |  |  |  |  |  |  |  |  |  |  |  |  |  |  |
|  | | 95 | 72 | 28 | 1.4 | 0.56 | -18.63 |  | 73 | 16 | 51 | 33 | 33 | 51 | 84 | 4.2 | 0.81 | 37.25 |
|  | | 96 | 9 | 91 | 4.55 | 6.60 | 44.38 |  | 76 | 26 | 46 | 28 | 28 | 46 | 74 | 3.7 | 0.81 | 27.25 |
|  | | 98 | 88 | 12 | 0.6 | 0.08 | -34.63 |  | 78 | 63 | 20 | 17 | 17 | 20 | 37 | 1.85 | 0.02 | -9.75 |
|  | | 102 | 35 | 65 | 3.25 | 3.31 | 18.38 |  | 80 | 94 | 4 | 2 | 2 | 4 | 6 | 0.3 | 0.01 | -40.75 |
|  | | 103 | 14 | 86 | 4.3 | 5.88 | 39.38 |  | 93 | 90 | 6 | 4 | 4 | 6 | 10 | 0.5 | 0.01 | -36.75 |
|  | | 115 | 83 | 17 | 0.85 | 0.19 | -29.63 |  | 101 | 78 | 13 | 9 | 9 | 13 | 22 | 1.1 | 0.04 | -24.75 |
|  | | 124 | 62 | 38 | 1.9 | 1.08 | -8.63 |  | 120 | 44 | 34 | 22 | 22 | 34 | 56 | 2.8 | 0.36 | 9.25 |
|  | | 128 | 64 | 36 | 1.8 | 0.96 | -10.63 |  | 121 | 15 | 54 | 31 | 31 | 54 | 85 | 4.25 | 1.32 | 38.25 |
| **Mean** | | | **53.38** | **46.63** | **2.33** | **2.33** | **0.00** |  |  | **53.25** | **28.50** | **18.25** | **18.25** | **28.50** | **46.75** | **2.34** | **0.42** | **0.00** |
|  | |  |  |  |  |  |  |  |  |  |  |  |  |  |  |  |  |  |
|  | |  |  |  |  |  |  |  |  |  |  |  |  |  |  |  |  |  |
|  | **Block** | | **Card 1** | **Card 1 +**  **Card 2** | **EV** | **Var.** | **Centered level of ambiguity** |  | **Block** | **Card 1** | **Card 2** | | **Card 3** | | **Card 1 +**  **Card 2** | **EV** | **Var.** | **Centered level of conflict** |
|  |  |  |  |  |  |  |  |  |  |  | **A** | **B** | **A** | **B** |  |  |  |  |
| Scan 3 | | |  |  |  |  |  |  |  |  |  |  |  |  |  |  |  |  |
|  | | 137 | 42 | 58 | 2.9 | 2.61 | 6.75 |  | 151 | 72 | 17 | 11 | 11 | 17 | 28 | 1.4 | 0.09 | -12.13 |
|  | | 142 | 16 | 84 | 4.2 | 5.60 | 32.75 |  | 153 | 84 | 10 | 6 | 6 | 10 | 16 | 0.8 | 0.04 | -24.13 |
|  | | 149 | 44 | 56 | 2.8 | 2.43 | 4.75 |  | 154 | 54 | 28 | 18 | 18 | 28 | 46 | 2.3 | 0.25 | 5.88 |
|  | | 150 | 75 | 25 | 1.25 | 0.44 | -26.25 |  | 155 | 29 | 44 | 27 | 27 | 44 | 71 | 3.55 | 0.72 | 30.88 |
|  | | 179 | 23 | 77 | 3.85 | 4.69 | 25.75 |  | 167 | 77 | 15 | 8 | 8 | 15 | 23 | 1.15 | 0.12 | -17.13 |
|  | | 181 | 39 | 61 | 3.05 | 2.90 | 9.75 |  | 171 | 76 | 15 | 9 | 9 | 15 | 24 | 1.2 | 0.09 | -16.13 |
|  | | 197 | 93 | 7 | 0.35 | 0.02 | -44.25 |  | 172 | 62 | 23 | 15 | 15 | 23 | 38 | 1.9 | 0.16 | -2.13 |
|  | | 198 | 58 | 42 | 2.1 | 1.33 | -9.25 |  | 192 | 25 | 47 | 28 | 28 | 47 | 75 | 3.75 | 0.90 | 34.88 |
| **Mean** | | | **48.75** | **51.25** | **2.56** | **2.50** | **0.00** |  |  | **59.88** | **24.88** | **15.25** | **15.25** | **24.88** | **40.13** | **2.01** | **0.30** | **0.00** |
|  | |  |  |  |  |  |  |  |  |  |  |  |  |  |  |  |  |  |
| Scan 4 | | |  |  |  |  |  |  |  |  |  |  |  |  |  |  |  |  |
|  | | 207 | 48 | 52 | 2.6 | 2.08 | -2.50 |  | 229 | 36 | 40 | 24 | 24 | 40 | 64 | 3.2 | 0.64 | 11.75 |
|  | | 210 | 60 | 40 | 2 | 1.20 | -14.50 |  | 230 | 86 | 8 | 6 | 6 | 8 | 14 | 0.7 | 0.01 | -38.25 |
|  | | 212 | 76 | 24 | 1.2 | 0.40 | -30.50 |  | 232 | 18 | 50 | 32 | 32 | 50 | 82 | 4.1 | 0.81 | 29.75 |
|  | | 214 | 30 | 70 | 3.5 | 3.85 | 15.50 |  | 236 | 46 | 33 | 21 | 21 | 33 | 54 | 2.7 | 0.36 | 1.75 |
|  | | 227 | 80 | 20 | 1 | 0.27 | -34.50 |  | 237 | 30 | 43 | 27 | 27 | 43 | 70 | 3.5 | 0.64 | 17.75 |
|  | | 235 | 26 | 74 | 3.7 | 4.32 | 19.50 |  | 249 | 38 | 38 | 24 | 24 | 38 | 62 | 3.1 | 0.49 | 9.75 |
|  | | 254 | 30 | 70 | 3.5 | 3.85 | 15.50 |  | 258 | 86 | 8 | 6 | 6 | 8 | 14 | 0.7 | 0.01 | -38.25 |
|  | | 255 | 14 | 86 | 4.3 | 5.88 | 31.50 |  | 262 | 42 | 36 | 22 | 22 | 36 | 58 | 2.9 | 0.49 | 5.75 |
| **Mean** | | | **45.5** | **54.5** | **2.73** | **2.73** | **0.00** |  |  | **47.75** | **32.00** | **20.25** | **20.25** | **32.00** | **52.25** | **2.61** | **0.43** | **0.00** |
|  | |  |  |  |  |  |  |  |  |  |  |  |  |  |  |  |  |  |
| **Overall mean** | | | | | **2.63** | **2.68** | **0.00** |  | | | | | | | | **2.49** | **0.44** | **0.00** |

**Note:** Ambiguity and conflict gambles were matched on expected value both overall and in each run (p > 0.52); due to a computational error, outcome variance under ambiguity was higher than under conflict (not equal as it was intended).

**S.2. Behavioral parameters**

**S.2.a Model free measures of risk, ambiguity and conflict attitudes.** Model free measures of risk, ambiguity, and conflict attitudes reflect general patterns in the individual choice data.

To define individual ambiguity (conflict) attitudes, we compared how often participants chose to bet on cards of type 2 and 3 under ambiguity (conflict) to how often they bet on these cards under risk (AA and CA respectively). If a participant choses to bet on these cards more often under risk than under ambiguity (conflict), we call this participant ambiguity (conflict) averse. If a participant choses to bet on these cards less often under risk than under ambiguity (conflict), we call this participant ambiguity (conflict) tolerant. An ambiguity (conflict) neutral participant will bet on cards 2 and 3 as often under ambiguity (conflict) as he or she does under risk.

$AA=\# of bets on cards 2 or 3 under risk-\# of bets on cards 2 or 3 under ambiguity$ (S.1.)

$CA=\# of bets on cards 2 or 3 under risk-\# of bets on cards 2 or 3 underconflict$ (S.2)

To define individual risk attitudes, we compared participants’ choices to choices of a hypothetical “risk-neutral” decision maker (the one who always choses an option that maximizes expected value). Because participants in our design had to choose among three options – sure gain, card 1, and card 2 or card 3 (always identical), the most natural way to define model free risk attitude is to compare how often participants chose a sure gain of $3 under risk to how often this option would be chosen by a risk neutral decision maker, which is 0 in our design (RA_SG_). If a participant choses to receive $3 for sure more often than a risk neutral decision maker, than we call this participant risk averse; if a participant choses to receive $3 for sure less often than a risk neutral decision maker, we call this participant risk tolerant. However, such measure will be derived very differently from model free measures of ambiguity and conflict attitudes (defined earlier). Therefore, we also employ a second measure of risk attitude (RA), defined similarly to our model free measures of ambiguity and conflict attitudes. That is, we compared how often participants chose to bet on cards of type 2 and 3 under risk to how often a risk neutral decision maker would do so (10 times in our design). If a participant choses to bet on these cards less often than a risk neutral decision maker, we call this participant risk averse. If a participant choses to bet on these cards more often than a risk neutral decision maker, we call this participant risk tolerant.

$RA_{SG}=how many times \$3 were chosen under risk$ (S.3)

$RA=10-\# of bets on cards 2 or 3 under risk$ (S.4)

Note that positive RASG, RA, AA, and CA reflect aversion, and negative – reflect tolerance.

Recall that for each participant 2 gambles from each condition were used twice. For these gambles we used an average of two choices. That is, if on one repetition a participant chose a sure payment of $3 and on another repetition the participant chose to bet on Card 1, we say that this participant chose $3 0.5 times, and chose to bet on Card 1 0.5 times.

**S.2.b Model based measures of ambiguity and conflict attitudes**

*Basic Model*

Choice behavior was modeled with a generalized multinomial logit model (Congdon, 2003). For each subject *i*,we define *p_ij_* as the probability that option j is chosen, where option j=1 is Type 1, option j=2 is Type 2 or Type 3, and option j=3 is a sure gain ($3); *p_ij_* is a function of the relative utility of the *j^th^* choice option:

*p_ij_* = $\frac{exp(U_{ij})}{\sum_{k} {exp(U}_{ik})}$ (S.5)

where $U_{ij}$ is the subjective expected utility of the j^th^ choice option for subject *i*.

Note that the resulting odds-model for comparing, say, the 2^nd^ with the 3^rd^ choice option is

$log\left( \frac{P(y_{i2} = 1)}{P(y_{i3} = 1)} \right)=U_{i2}-U_{i3}$ , (S.6)

which is a natural comparative model.

More specifically, we model the utility function the following way.

For j=1,

$U_{i1}=\lambda W_{i1}$, (S.7)

for j > 1,

$U_{ij}=\beta_{j}+\lambda W_{ij}$, (S.8)

where W_ij_ incorporates the subjective monetary and probabilistic judgments of the j^st^ choice option that predict its likelihood of being chosen by i^th^ person, the $\beta_{j}$ are parameters that tap into any bias towards or away from the j^th^ choice option (j>1) that are not determined by money or subjective probability, because $U_{i2}-U_{i3}=\beta_{2}-\beta_{3}+\lambda\left( W_{i2}-W_{i3} \right)$.

Further,

$W_{ij}=x_{j}^{\theta_{i}}\pi_{j}^{\gamma_{ij}}$, (S.9)

where $x_{j}$ is the monetary payoff for the j^th^ choice option (j=1 for sure gain, j=2 for Type 1, j=3 for Type 2 or Type 3), π_j_= is the winning probability of that payoff, $\theta_{i}$ is subject i’s monetary utility parameter, and $\gamma_{ij}$ is subject i’s probability weighting parameter for the j^th^ task condition (j=1 for ambiguity gambles, j=2 for conflict gambles, and j=3 for ignorance gambles; for all risk gambles $\gamma_{i}=1$, to make the model identifiable).

To test a hypothesis that the probability weighting parameter differ across task conditions where the probabilities are not fully known (i.e., ambiguity, conflict, and ignorance), we have compared a model for which the $\gamma_{ij}$ are identical across those conditions (Model 1) against one where they may differ (Model 2). Table S.4.b.1.shows how the $x_{j}^{\theta_{i}}\pi_{j}^{\gamma_{ij}}$are set up for these two models. In Model 1 γ = γ in the Type 2 or 3 choice option for all conditions, whereas in Model 2 γ = γ__ γ__ or γ__ depending on the condition.

*Interpreting the Parameters Relative to a “Rational” Agent*

The  parameter is a monetary utility parameter in the sense that it indicates whether a person’s utility for money is convex (discounting larger values) or concave (inflating larger values).  < 1 implies a convex function and  > 1 a concave function.

The γ parameter indicates whether a person is “optimistic” or “pessimistic” regarding the expected values of unknown probabilities. γ > 1 implies that they are pessimistic because it deflates the probabilities, whereas γ < 1 implies optimism. For a “rational” expected utility (EU) agent  = 1 and γ = 1.

The winning probability associated with option 2 (π_2_) in both the conflict and ambiguity conditions was defined as the average number of Type 2 and Type 3 cards, divided by the total number of cards (100). Note, however, that the intuition behind this expression was different for ambiguity and for conflict. Under ambiguity, to derive the winning probability, we followed an *ignorance prior* hypothesis. Consider, for instance, the case depicted in figure 1 A-ii (main text). The total number of Type 2 and Type 3 cards is equal to 94, which means that the “true” probability of drawing Card 2 from the mixed deck is between 1/100 and 93/100. An *ignorance prior* hypothesis suggests that all these probabilities should have the same subjective weighting for an ambiguity neutral decision maker. Consequently, an expected probability of drawing a Type 2 card from the mixed deck in this case is equal to (0.01 +0.93)/2, or ((Type 2 + Type 3)/2)/100. Same holds for Type 3. Under conflict, we assumed that if a conflict neutral decision maker does not have any reason to trust one source more than the other (A versus B), then he/she should weigh both opinions equally. Consider, for instance, the case depicted in figure 1 B-ii. The expected winning probability associated with Type 2 is equal to (0.5*0.58 + 0.5*0.36), or yet again ((Type 2 + Type 3)/2)/100. Same holds for Type 3. This also implies that expected value of betting on Type 2 or Type 3 cards is the same under ambiguity and conflict.

*Refinements and Final Models*

The final version of our models requires that we take into account any effects of display orientation (the right- versus left-oriented display), and potential gender differences, in view of the large empirical literature suggesting that males and females might be susceptive to different biases in decision making.

Finally, the fact that there is a majority of Type 1 choices suggests that there may be a bias towards Type 1 that is not accounted for by either monetary or probabilistic assessments. Therefore we have introduced a β__ term into the models to test for this.

Let G denote gender and D display orientation. Let j = 3 🡪 sure payoff, j = 2 🡪 uncertain alternative (Type 2 or 3), and j = 1 🡪 risky alternative (Type 1). Likewise, for the uncertainty conditions let k = 1 🡪 ambiguity, k = 2 🡪 conflict, k = 3 🡪 ignorance, and k = 4 🡪 risk. Then we can write the U_ij_ terms for the general model as follows:

$\begin{matrix} U_{i1}= \beta_{0}+\beta_{g}G_{i}+\beta_{d}D_{i}+\lambda_{i}{10}^{\theta_{i}}\pi_{1} \\ U_{i2}=\lambda_{i}{10}^{\theta_{i}}\pi_{2}^{\gamma_{ik}}, with the restriction that \gamma_{i4} = 1 \\ U_{i3}=\lambda_{i}3^{\theta_{i}} \end{matrix}$ (S.10)

For this paper the main parameters of interest are γ_i1 (_an individual attitude toward ambiguity),andγ_i2_ (an individual attitude toward conflict). The model also includes three dummy parameters: gender (G, male or female), display orientation (D, gamble is on the right or gamble is on the left), and the intercept, (β0), that taps into any bias towards or away from the j^th^ alternative that are not determined by money or subjective probability. These parameters are included in the known-probability (risky) choice option utility formula because this condition is used as a reference in the model. λ reflects an individual sensitivity of choice probability to the degree of inflection of utility difference, or the amount of “randomness” in the participant’s choices (λ=0 implies random choices; as λ increases the function is more steeply inflected at zero).

Model 2 differs from Model 1 only in the γ exponent for U_i2_. Model 1 imposes the restriction that γ__ = γ__ = γ__ = γ__

**S.3. Behavioral data**

**S.3.a. Response Time by Uncertain Conditions.** We compared response times under risk, ambiguity, and conflict, using a paired t test. Response time under risk was significantly shorter than under ambiguity (t (31)=-3.139, p<0.01) or conflict (t (31)=-4.714, p<0.01). Response time under ambiguity did not differ significantly from response time under conflict (t(31)=-1.077, p=0.29).

*Table S.3.a.Average response times for each participant under risk, ambiguity, and conflict.*

| **subject** | **Risk** | **Ambiguity** | **Conflict** |
| --- | --- | --- | --- |
| 1 | 2.76 | 3.44 | 3.26 |
| 2 | 2.11 | 2.20 | 2.20 |
| 3 | 2.10 | 1.85 | 2.23 |
| 4 | 1.86 | 2.00 | 2.53 |
| 5 | 1.66 | 1.88 | 1.70 |
| 6 | 1.64 | 1.39 | 1.54 |
| 8 | 3.28 | 3.19 | 3.41 |
| 10 | 2.32 | 2.59 | 2.43 |
| 11 | 2.20 | 2.27 | 2.16 |
| 12 | 2.59 | 2.89 | 2.97 |
| 13 | 2.35 | 2.50 | 2.79 |
| 14 | 1.66 | 1.78 | 2.32 |
| 15 | 1.70 | 1.70 | 1.72 |
| 16 | 2.38 | 3.19 | 2.60 |
| 17 | 1.50 | 1.84 | 1.70 |
| 19 | 2.10 | 2.68 | 2.37 |
| 20 | 2.43 | 2.10 | 2.37 |
| 22 | 1.83 | 2.16 | 2.42 |
| 23 | 2.55 | 2.75 | 2.66 |
| 24 | 2.29 | 2.61 | 2.50 |
| 25 | 2.29 | 2.89 | 2.25 |
| 26 | 2.02 | 2.16 | 2.36 |
| 27 | 2.53 | 2.90 | 2.94 |
| 30 | 2.09 | 2.06 | 2.32 |
| 33 | 2.22 | 2.60 | 2.41 |
| 34 | 2.59 | 2.65 | 2.90 |
| 35 | 3.38 | 2.87 | 2.73 |
| 36 | 1.83 | 1.81 | 2.08 |
| 38 | 2.08 | 2.28 | 2.51 |
| 39 | 3.40 | 3.68 | 3.63 |
| 41 | 2.00 | 2.03 | 2.14 |
| 42 | 1.85 | 1.77 | 2.32 |
| **average** | **2.24** | **2.40** | **2.45** |

**S.3.b. Choice Frequencies by Uncertain Conditions.**

*Table S.3.b.1. Choice frequencies under risk.*

| **subject** | **SG** | **Card 2 + Card 3** | **Card 1** | **missing** |
| --- | --- | --- | --- | --- |
| 1 | 0.17 | 0.20 | 0.60 | 0.03 |
| 2 | 0.67 | 0.03 | 0.30 | 0.00 |
| 3 | 0.13 | 0.23 | 0.63 | 0.00 |
| 4 | 0.10 | 0.27 | 0.63 | 0.00 |
| 5 | 0.33 | 0.03 | 0.63 | 0.00 |
| 6 | 0.10 | 0.35 | 0.55 | 0.00 |
| 8 | 0.28 | 0.28 | 0.43 | 0.00 |
| 10 | 0.38 | 0.10 | 0.52 | 0.00 |
| 11 | 0.13 | 0.23 | 0.63 | 0.00 |
| 12 | 0.18 | 0.20 | 0.62 | 0.00 |
| 13 | 0.00 | 0.33 | 0.67 | 0.00 |
| 14 | 0.00 | 0.33 | 0.67 | 0.00 |
| 15 | 0.47 | 0.07 | 0.47 | 0.00 |
| 16 | 0.40 | 0.00 | 0.43 | 0.17 |
| 17 | 0.37 | 0.07 | 0.57 | 0.00 |
| 19 | 0.37 | 0.10 | 0.53 | 0.00 |
| 20 | 0.60 | 0.03 | 0.37 | 0.00 |
| 22 | 0.38 | 0.05 | 0.57 | 0.00 |
| 23 | 0.07 | 0.27 | 0.67 | 0.00 |
| 24 | 0.30 | 0.13 | 0.57 | 0.00 |
| 25 | 0.40 | 0.07 | 0.50 | 0.03 |
| 26 | 0.48 | 0.05 | 0.47 | 0.00 |
| 27 | 0.00 | 0.23 | 0.53 | 0.23 |
| 30 | 0.20 | 0.20 | 0.60 | 0.00 |
| 33 | 0.67 | 0.00 | 0.30 | 0.03 |
| 34 | 0.12 | 0.22 | 0.63 | 0.03 |
| 35 | 0.23 | 0.17 | 0.57 | 0.03 |
| 36 | 0.00 | 0.33 | 0.67 | 0.00 |
| 38 | 0.60 | 0.30 | 0.10 | 0.00 |
| 39 | 0.07 | 0.37 | 0.57 | 0.00 |
| 41 | 0.00 | 0.33 | 0.67 | 0.00 |
| 42 | 0.20 | 0.13 | 0.67 | 0.00 |
| **average** | **0.26** | **0.18** | **0.54** | **0.02** |
| **risk neutral** | **0** | **0.31** | **0.69** | **0** |

*Table S.3.b.2. Choice frequencies under ambiguity.*

| **subject** | **SG** | **Card 2 + Card 3** | **Card 1** | **missing** |
| --- | --- | --- | --- | --- |
| 1 | 0.20 | 0.30 | 0.47 | 0.03 |
| 2 | 0.73 | 0.00 | 0.27 | 0.00 |
| 3 | 0.37 | 0.00 | 0.63 | 0.00 |
| 4 | 0.20 | 0.20 | 0.60 | 0.00 |
| 5 | 0.28 | 0.22 | 0.50 | 0.00 |
| 6 | 0.00 | 0.47 | 0.53 | 0.00 |
| 8 | 0.17 | 0.37 | 0.47 | 0.03 |
| 10 | 0.27 | 0.27 | 0.47 | 0.00 |
| 11 | 0.17 | 0.37 | 0.47 | 0.00 |
| 12 | 0.07 | 0.40 | 0.53 | 0.00 |
| 13 | 0.00 | 0.40 | 0.60 | 0.00 |
| 14 | 0.00 | 0.33 | 0.67 | 0.00 |
| 15 | 0.40 | 0.20 | 0.40 | 0.00 |
| 16 | 0.47 | 0.00 | 0.43 | 0.09 |
| 17 | 0.32 | 0.18 | 0.50 | 0.00 |
| 19 | 0.35 | 0.15 | 0.50 | 0.00 |
| 20 | 0.48 | 0.07 | 0.38 | 0.06 |
| 22 | 0.20 | 0.33 | 0.43 | 0.03 |
| 23 | 0.00 | 0.37 | 0.63 | 0.00 |
| 24 | 0.30 | 0.33 | 0.37 | 0.00 |
| 25 | 0.27 | 0.27 | 0.43 | 0.03 |
| 26 | 0.03 | 0.50 | 0.47 | 0.00 |
| 27 | 0.00 | 0.33 | 0.53 | 0.13 |
| 30 | 0.08 | 0.35 | 0.57 | 0.00 |
| 33 | 0.43 | 0.30 | 0.27 | 0.00 |
| 34 | 0.13 | 0.20 | 0.53 | 0.13 |
| 35 | 0.18 | 0.25 | 0.47 | 0.09 |
| 36 | 0.00 | 0.42 | 0.58 | 0.00 |
| 38 | 0.57 | 0.27 | 0.17 | 0.16 |
| 39 | 0.00 | 0.40 | 0.60 | 0.00 |
| 41 | 0.00 | 0.55 | 0.45 | 0.00 |
| 42 | 0.13 | 0.37 | 0.50 | 0.00 |
| **average** | **0.21** | **0.29** | **0.48** | **0.02** |
| **ambiguity neutral**  *(group average under risk)* | **0.26** | **0.18** | **0.54** | **0.02** |

*Table S.3.b.3. Choice frequencies under conflict.*

| **subject** | **SG** | **Card 2 + Card 3** | **Card 1** | **missing** |
| --- | --- | --- | --- | --- |
| 1 | 0.30 | 0.13 | 0.57 | 0.00 |
| 2 | 0.60 | 0.00 | 0.37 | 0.03 |
| 3 | 0.27 | 0.10 | 0.60 | 0.03 |
| 4 | 0.15 | 0.22 | 0.63 | 0.00 |
| 5 | 0.40 | 0.03 | 0.57 | 0.00 |
| 6 | 0.00 | 0.37 | 0.63 | 0.00 |
| 8 | 0.17 | 0.30 | 0.50 | 0.03 |
| 10 | 0.48 | 0.00 | 0.52 | 0.00 |
| 11 | 0.00 | 0.33 | 0.67 | 0.00 |
| 12 | 0.07 | 0.20 | 0.70 | 0.03 |
| 13 | 0.00 | 0.25 | 0.75 | 0.00 |
| 14 | 0.00 | 0.33 | 0.67 | 0.00 |
| 15 | 0.50 | 0.00 | 0.50 | 0.00 |
| 16 | 0.47 | 0.00 | 0.40 | 0.13 |
| 17 | 0.43 | 0.03 | 0.53 | 0.00 |
| 19 | 0.40 | 0.07 | 0.50 | 0.03 |
| 20 | 0.53 | 0.00 | 0.43 | 0.03 |
| 22 | 0.38 | 0.08 | 0.47 | 0.07 |
| 23 | 0.07 | 0.27 | 0.63 | 0.03 |
| 24 | 0.48 | 0.02 | 0.47 | 0.03 |
| 25 | 0.60 | 0.00 | 0.37 | 0.03 |
| 26 | 0.30 | 0.20 | 0.50 | 0.00 |
| 27 | 0.03 | 0.23 | 0.57 | 0.17 |
| 30 | 0.25 | 0.22 | 0.53 | 0.00 |
| 33 | 0.53 | 0.07 | 0.37 | 0.03 |
| 34 | 0.20 | 0.17 | 0.53 | 0.10 |
| 35 | 0.43 | 0.00 | 0.47 | 0.10 |
| 36 | 0.00 | 0.33 | 0.67 | 0.00 |
| 38 | 0.58 | 0.18 | 0.23 | 0.00 |
| 39 | 0.12 | 0.35 | 0.50 | 0.03 |
| 41 | 0.00 | 0.22 | 0.75 | 0.03 |
| 42 | 0.23 | 0.03 | 0.73 | 0.00 |
| **average** | **0.28** | **0.15** | **0.54** | **0.03** |
| **conflict neutral**  *(group average under risk)* | **0.26** | **0.18** | **0.54** | **0.02** |

**S.3.c. Model free measures of risk, ambiguity and conflict attitudes**

Majority of our participants were very consistent in their choices. Only 4 participants changed their choice on two repetitions, 16 participants changed their choice on 1 repetition. Six of these changes occurred under risk, 8 under ambiguity, and 9 under conflict.

Two participants (3 and 26) were outliers in their ambiguity and conflict attitudes, and were excluded from the further analyses (see figures S.4.a).

On a group level, participants demonstrated risk and conflict aversion, but ambiguity seeking. Two measures of risk attitudes significantly correlated (r = 0.768, p < 0.001), which suggests that both measures provide similar information about individual risk attitudes, and that either measure can be used to quantify individual risk attitudes. Risk, ambiguity, and conflict attitudes did not significantly correlate (p>0.10), which suggests that risk, ambiguity, and conflict are orthogonal measures.

*Table S.3.c.1. Model free attitudes toward risk, ambiguity, and conflict*

| **subject** | **RA_SG_** | **RA** | **AA** | **CA** |
| --- | --- | --- | --- | --- |
| 1 | 5 | 4 | -3 | 2 |
| 2 | 20 | 9 | 1 | 1 |
| **3** | 4 | 3 | **7** | 4 |
| 4 | 3 | 2 | 2 | 1.5 |
| 5 | 10 | 9 | -5.5 | 0 |
| 6 | 3 | -0.5 | -3.5 | -0.5 |
| 8 | 8.5 | 1.5 | -2.5 | -0.5 |
| 10 | 11.5 | 7 | -5 | 3 |
| 11 | 4 | 3 | -4 | -3 |
| 12 | 5.5 | 4 | -6 | 0 |
| 13 | 0 | 0 | -2 | 2.5 |
| 14 | 0 | 0 | 0 | 0 |
| 15 | 14 | 8 | -4 | 2 |
| 16 | 12 | 10 | 0 | 0 |
| 17 | 11 | 8 | -3.5 | 1 |
| 19 | 11 | 7 | -1.5 | 1 |
| 20 | 18 | 9 | -1 | 1 |
| 22 | 11.5 | 8.5 | -8.5 | -1 |
| 23 | 2 | 2 | -3 | 0 |
| 24 | 9 | 6 | -6 | 3.5 |
| 25 | 12 | 8 | -6 | 2 |
| **26** | 14.5 | 8.5 | **-13.5** | **-4.5** |
| 27 | 0 | 3 | -3 | 0 |
| 30 | 6 | 4 | -4.5 | -0.5 |
| 33 | 20 | 10 | -9 | -2 |
| 34 | 3.5 | 3.5 | 0.5 | 1.5 |
| 35 | 7 | 5 | -2.5 | 5 |
| 36 | 0 | 0 | -2.5 | 0 |
| 38 | 18 | 1 | 1 | 3.5 |
| 39 | 2 | -1 | -1 | 0.5 |
| 41 | 0 | 0 | -6.5 | 3.5 |
| 42 | 6 | 6 | -7 | 3 |

*Figure S.3.c.1. Normal Q-Q Plot of Ambiguity Attitudes*

**
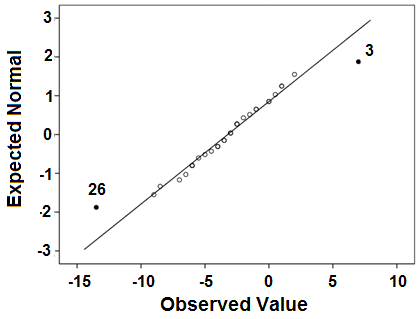
**

*Figure S.3.c.2. Boxplot of ambiguity attitudes*

**
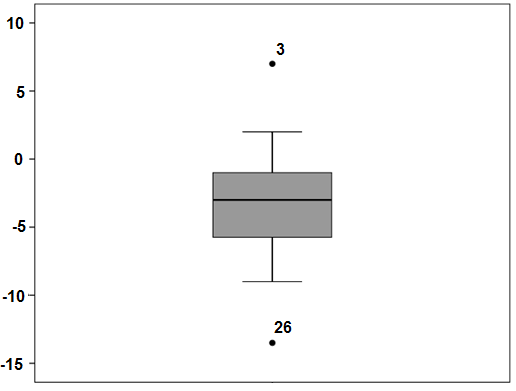
**

*Figure S.3.c.3. Normal Q-Q Plot of Conflict Attitudes*

**
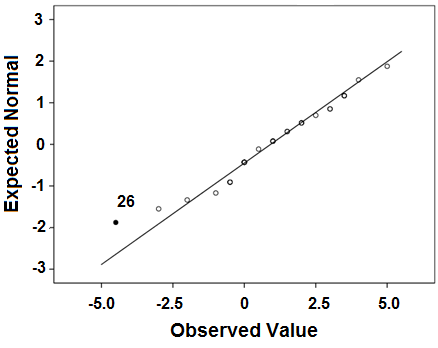
**

*Figure S.3.c.4. Boxplot of Conflict Attitudes*

**
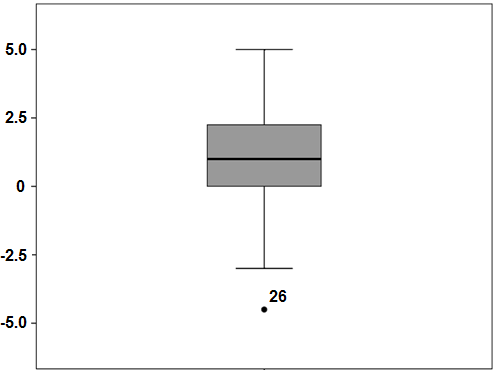
**

*Table. S.3.c.2. Group level attitudes toward risk, ambiguity, and conflict (N=30).*

|  | **Descriptive statistics** | | **t-test for mean being different from 0** | | **Correlations** | | | |
| --- | --- | --- | --- | --- | --- | --- | --- | --- |
|  | **Mean** | **Std. Error** | **t (29)** | **p** | **RA_SG_** | **RA** | **AA** | **CA** |
| **RA_SG_** | 7.78 | 1.13 | 6.907 | .000 | 1 |  |  |  |
| **RA** | 4.57 | 0.65 | 7.028 | .000 | **.768^*^** | 1 |  |  |
| **AA** | -3.22 | 0.52 | -6.198 | .000 | -.076 | -.308 | 1 |  |
| **CA** | 1.00 | 0.32 | 3.084 | .004 | .028 | -.042 | .128 | 1 |

Note: **^*^** - significance at the p<0.001

*Figure S.3.c.5. Scatter plots of risk, ambiguity, and conflict attitudes (N=30).*


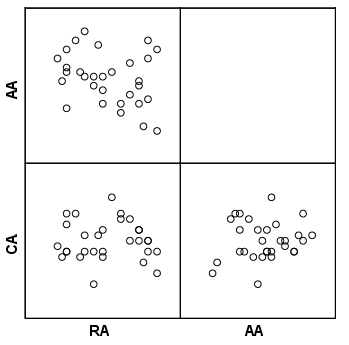


**S.3.d. Model based analysis of the choice data**

*Overall Patterns and Model Fit.*

To estimate the model parameters we used Bayesian MCMC methods in WinBUGs v. 1.4. The observed choice probabilities are displayed in the upper part of Table S.4.b.2 for each uncertainty condition. An obvious pattern is that the ambiguity and ignorance conditions resemble one another, while the conflict and risk conditions are similar. People are generally more inclined to choose Type 2 or 3 under the ambiguity and ignorance, and correspondingly less inclined to choose Type 1 or the Sure Payoff. These impressions are borne out in the analysis, although the picture is more complex than this when it comes to individual differences in choice-patterns.

The middle part of Table S.4.b.2 shows the predicted probabilities from Model 2, and the lower part displays the root-mean-squared-errors (RMSE) for this model. It can be seen that although the model fits the data reasonably well (the total RMSE is about .045), it does not fit the Risk condition as well as the other three. The fit also is quite good for all subjects except one (subject 26, RMSE = .084; recall that subject 26 was also classified as an outliers by the model free measures). Figure S.4.b.1shows the plot of observed versus fitted choice probabilities, with the outlier points due to subject 26.

*Parameter Estimates*

Model 2 parameter estimates are summarized in Table S.4.b.3. Starting with the “nuisance” parameters, the β1 estimate is well above 0, reflecting a bias towards choosing Type 1 for reasons not attributable to monetary or probability judgments. The β_G_ parameter’s 95% credible interval nearly excludes 0, suggesting a tendency for males to choose Type 1 more often than females do, but not a strong difference. Likewise, the parameter β_D_ is quite close to 0, indicating no effect from display orientation on the choice probabilities. Finally, the distribution of  values is considerably dispersed, but all of them are well above 0 (i.e., all of their 95% credibility intervals exclude 0), implying that none of the subjects has been choosing “randomly.” The average  is well below 1, and in fact 29 subjects’  values were below 1, replicating the well-known finding that most people have concave monetary utility functions. The remaining four subjects do not exceed 1 by much (the highest  is 1.216).

Table *S.4.b.1*. $W_{ij}$ Parameterization for Two Models

Choice option Risk Ambiguity Conflict Ignorance

Sure payoff 3^^ 3^^ 3^^ 3^^

Card 1 10^^ 10^^ 10^^ 10^^

Model 1:

Card 2 or 3 10^^ 10^^^γ^ 10^^^γ^ 10^^^γ^

Model 2:

Card 2 or 3 10^^ 10^^^^ 10^^^^ 10^^^^

Table *S.4.b2.* Choice Probabilities by Uncertainty Condition

Observed Probabilities

|  | Card1 | Card 2 or 3 | Sure Payoff |
| --- | --- | --- | --- |
| Ambiguity | 0.48 | 0.29 | 0.21 |
| Conflict | 0.54 | 0.15 | 0.28 |
| Ignorance | 0.48 | 0.31 | 0.20 |
| Risk | 0.54 | 0.18 | 0.26 |

Model 2 Predicted Probabilities

|  | Card1 | Card 2 or 3 | Sure Payoff |
| --- | --- | --- | --- |
| Ambiguity | 0.53 | 0.28 | 0.19 |
| Conflict | 0.57 | 0.19 | 0.25 |
| Ignorance | 0.53 | 0.29 | 0.18 |
| Risk | 0.56 | 0.23 | 0.21 |

Model 2 RMSE

|  | Card1 | Card 2 or 3 | Sure Payoff |
| --- | --- | --- | --- |
| Ambiguity | 0.033 | 0.028 | 0.037 |
| Conflict | 0.039 | 0.029 | 0.048 |
| Ignorance | 0.039 | 0.033 | 0.047 |
| Risk | 0.059 | 0.072 | 0.056 |

Table *S.4.b.3.*  Model 2 Parameter and Mean Parameter Estimates

|  |  |  | Credible | Interval |
| --- | --- | --- | --- | --- |
| parameters | mean | sd | 2.50% | 97.50% |
| *β_1_* | 3.375 | 0.239 | 2.907 | 3.846 |
| *β_G_* | 0.515 | 0.271 | -0.008 | 1.051 |
| *β_D_* | -0.083 | 0.267 | -0.587 | 0.444 |
| *λ* | 5.650 | 0.218 | 5.223 | 6.085 |
| *θ* | 0.377 | 0.019 | 0.342 | 0.417 |
| *γ_A_* | 0.854 | 0.020 | 0.850 | 1.082 |
| *γ_C_* | 1.688 | 0.160 | 1.481 | 1.870 |
| *γ__^^* | 0.882 | 0.053 | 0.778 | 0.987 |
| *γ_C_ – γ_A_* | 0.834 | 0.106 | 0.601 | 0.921 |
| *γ__ – γ_A_* | -0.028 | 0.070 | -0.222 | 0.055 |

^*^ - *γ__ *− denotes a probability waiting parameter for sample space ignorance, *γ__* This condition is discussed in greater details in Pushkarskaya et al., 2010).

*Figure S.4.b.1. Scatter plot of observed versus fitted choice probabilities via Model 2.*


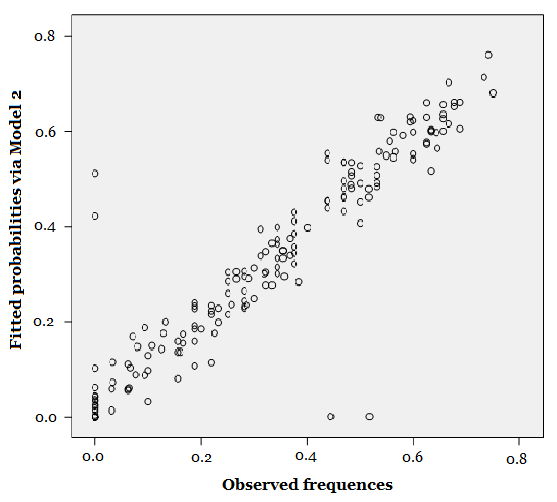


**S.4. Participants split for out-of-sample confirmation analysis**

Two groups were matched on age, gender, and behavioral preferences.

Table *S.4.* Individual characteristics of participants by groups

|  | **Group 1**  **(N = 15)** | **Group 2**  **(N = 15)** | **Pooled sample**  **(N = 30)** | **F(1,28)** | **** |
| --- | --- | --- | --- | --- | --- |
| **Age** | 27.20 | 24.33 | 25.77 | 1.88 | 0.18 |
| **Female** | 0.53 | 0.60 | 0.57 | 0.13 | 0.72 |
| **γ_A_** | 0.82 | 0.68 | 0.75 | 0.67 | 0.42 |
| **ln γ_A_** | -0.16 | -0.21 | -0.18 | 0.44 | 0.51 |
| **γ_C_** | 1.77 | 1.62 | 1.69 | 0.22 | 0.64 |

**S.5. Additional fMRI statistical maps.**

**Figure S.5.a. Categorical effect of ambiguity (Ambiguity > Conflict) and conflict (Conflict > Risk) on activation patterns.**

**
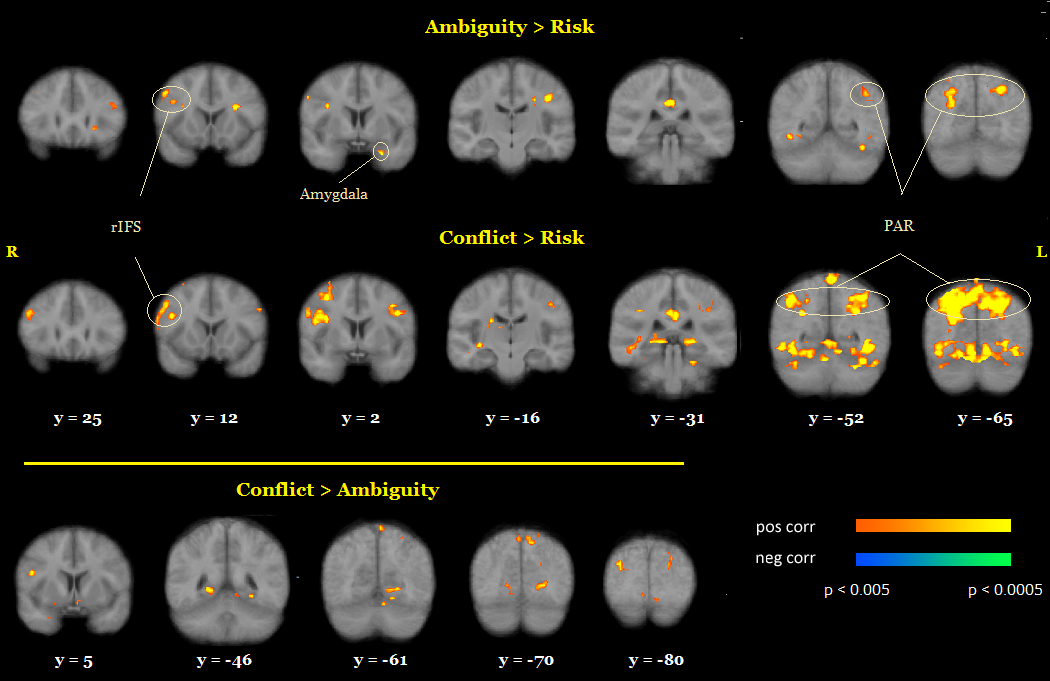
**

**Note:** Increased activation under ambiguity compared to risk was found in the right inferior frontal sulcus (rIFS; x=41 y=12 z=30, neighboring to Huettel et al. (2006) x=38 y = 17 z=32) of lateral prefrontal cortex, in the left amygdala (x=-23 y=1 z=-21, neighboring to Hsu et al. (2005) x=-21 y=-8 z = -12), and broad areas in the posterior parietal cortex (PAR; similarly to Rustichini et al. (2005), Huettel et al. (2006), Bach et al. (2009), and others); all coordinates are Talairach coordinates.

**Figure S.5.b. Correlation of ambiguity (Ambiguity > Conflict) and conflict (Conflict > Risk) effects with behavioral preferences.**

**
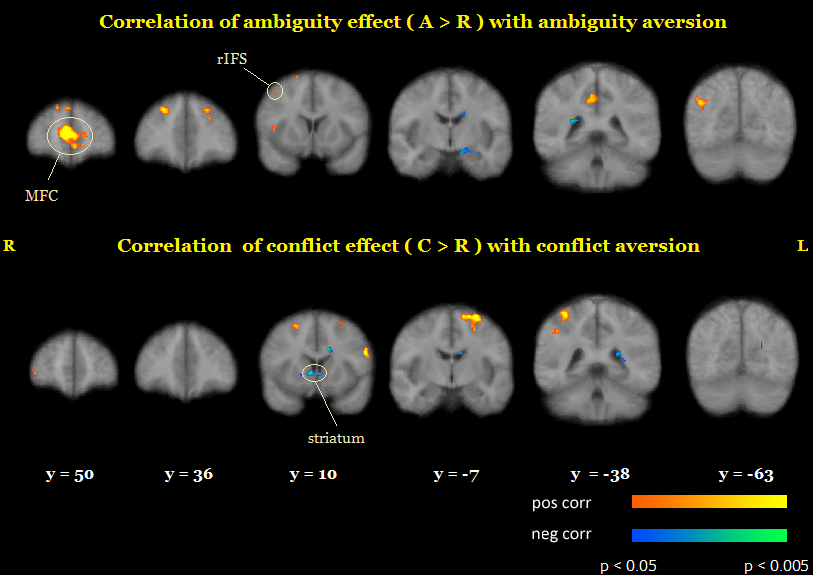
**

**Note:** Ambiguity effect compared to riskcorrelated positively with ambiguity aversion across participants in the right inferior frontal sulcus (rIFS; x=41 y=10 z=48, neighboring to Huettel et al. (2006) x=40 y = 8 z=42) and in the medial frontal cortex (MFC); conflict effect compared to risk correlated negatively with conflict aversion across participants in the ventral striatum; all coordinates are Talairach coordinates.

**Table S.6. List of regions by fMRI statistical maps (full brain search, pooled sample N=30).**

| **#** | | **ROI** | **BA** | **x** | **y** | **z** | **size** |
| --- | --- | --- | --- | --- | --- | --- | --- |
| Ambiguity level (p < 0.01) | | | | | | | |
| 1 | | Ventromedial Prefrontal Cortex / Anterior Cingulate **( - )** | 24 | 3 | 23 | -7 | 968 |
| 2 | | Right Insula **( - )** | 13 | 35 | 4 | 3 | 1260 |
| 3 | | Right Insula **( - )** | 13 | 41 | -10 | 2 | 2023 |
| 4 | | Paracentral Lobule **( - )** | 31 | 8 | -30 | 47 | 1054 |
| 5 | | Right Parietal Lobule **( - )** | 40 | 54 | -38 | 27 | 2026 |
| 6 | | Right Parietal Lobule **( + )** | 19 | 32 | -65 | 36 | 2878 |
| 7 | | Parietal Lobe **( + )** | 7 | 0 | -63 | 64 | 999 |
|  | |  |  |  |  |  |  |
| Conflict level (p < 0.01) | | | | | | | |
| 1 | | Left Striatum **( + )** |  | -5 | 9 | -3 | 148 |
| 2 | | Right Insula **( - )** | 40 | 47 | -23 | 16 | 573 |
| 3 | | Right Precentral Gyrus **( - )** | 4 | 13 | -27 | 64 | 179 |
| 4 | | Left Middle Temporal Gyrus **( + )** | 20 | -47 | -32 | -8 | 175 |
| 5 | | Right Middle Temporal Gyrus **( - )** | 37 | 52 | -60 | 3 | 522 |
| 6 | | Left Parietal Lobule **( + )** | 40 | -32 | -43 | 32 | 544 |
| 7 | | Right Occipital Lobe **( + )** | 18 | 22 | -78 | 1 | 655 |
| 8 | | Left Cerebellum **( + )** |  | -6 | -65 | -11 | 151 |
| 9 | | Left Cerebellum **( + )** |  | -32 | -71 | -21 | 315 |
|  | |  |  |  |  |  |  |
| **#** | | **ROI** | **BA** | **x** | **y** | **z** | **size** |
| Conflict level > Ambiguity Level (p < 0.01) | | | | | | | |
| 1 | | Ventromedial Prefrontal Cortex / Anterior Cingulate **( + )** | 25 | 4 | 13 | -7 | 214 |
| 2 | | Left Striatum **( + )** |  | -6 | 12 | -6 | 242 |
| 3 | | Left Middle Frontal Gyrus **( - )** | 9 | -36 | 50 | 28 | 259 |
| 4 | | Left Inferior Frontal Gyrus **( + )** | 13 | -32 | 6 | -12 | 504 |
| 5 | | Right Insula **( + )** |  | 39 | -11 | 0 | 539 |
| 6 | | Left Temporal Lobe **( - )** | 37 | -54 | -54 | -15 | 260 |
| 7 | | Right Occipital Lobe **( + )** | 17 | 20 | -80 | 6 | 552 |
|  | |  |  |  |  |  |  |
| Ambiguity main effect correlated with ambiguity aversion  (Random Effects ANCOVA, p< 0.05) | | | | | | | |
| 1 | | Medial Frontal Gyrus **( + )** | 9 | 3 | 53 | 17 | 463 |
| 2 | | Cingulate Gyrus **( + )** | 31 | 3 | -40 | 38 | 145 |
| 3 | | Middle Temporal Gyrus **( + )** | 39 | 39 | -64 | 30 | 369 |
|  | |  |  |  |  |  |  |
| Conflict main effect correlated with conflict aversion  (Random Effects ANCOVA, p< 0.05) | | | | | | | |
| 1 | | Right Ventral Striatum **( - )** |  | 8 | 13 | 5 | 397 |
| 2 | | Right Superior Temporal **( + )** | 38 | 43 | 8 | -17 | 174 |
| 3 | | Left Middle Frontal Gyrus **( + )** | 6 | -26 | -12 | 60 | 171 |
| 4 | | Right Inferior Parietal Lobule **( + )** | 40 | 34 | -40 | 54 | 2535 |
| 5 | | Right Parietal Lobe **( + )** | 3 | 41 | -24 | 58 | 314 |
| **#** | | **ROI** | **BA** | **x** | **y** | **z** | **size** |
| 6 | | Right Paracentral Lobule **( + )** | 5 | 1 | -31 | 55 | 197 |
| 7 | | Right Posterior Cingulate **( + )** | 30 | 3 | -59 | 12 | 235 |
| Ambiguity effect compared to Risk (A > R, p < 0.005) | | | | | | | |
| 1 | | Left Anterior Cingulate **( + )** | 32 | -24 | 34 | 10 | 329 |
| 2 | | Left Anterior Insula **( + )** |  | -22 | 25 | 2 | 95 |
| 3 | | Right Middle Frontal Gyrus **( + )** | 8 | 48 | 11 | 39 | 898 |
| 4 | | Left Middle Frontal Gyrus **( + )** | 9 | -32 | 17 | 26 | 1326 |
| 5 | | Left Middle Frontal Gyrus **( + )** | 11 | -38 | 35 | -18 | 569 |
| 6 | | Right Inferior Frontal Gyrus **( + )** | 9 | 37 | 8 | 30 | 445 |
| 7 | | Left Amygdala **( + )** |  | -23 | 1 | -21 | 92 |
| 8 | | Left Cingulate Gyrus **( + )** | 32 | -2 | -32 | 30 | 1196 |
| 9 | | Right Inferior Parietal Lobule **( + )** | 40 | 38 | -51 | -6 | 502 |
| 10 | | Left Inferior Parietal Lobule **( + )** | 40 | -40 | -51 | 37 | 298 |
| 11 | | Left Culmen **( + )** |  | -38 | -55 | -18 | 821 |
| 12 | | Left Middle Temporal Gyrus **( + )** | 37 | -40 | -56 | -4 | 287 |
| 13 | | Right Parietal Lobe **( + )** | 7 | 7 | -74 | 47 | 1954 |
| 14 | | Left Parietal Lobe **( + )** | 7 | -26 | -65 | 39 | 1825 |
| 15 | | Left Parietal Lobe **( + )** | 7 | -28 | -67 | 32 | 2616 |
|  | |  |  |  |  |  |  |
| Conflict effect compared to Risk (C > R, p < 0.005) | | | | | | | |
| 1 | | Right Middle Frontal Gyrus **( + )** | 9 | 45 | 10 | 32 | 6604 |
| 2 | | Right Middle Frontal Gyrus **( + )** | 6 | 35 | -1 | 49 | 6297 |
| **#** | **ROI** | | **BA** | **x** | **y** | **z** | **size** |
| Conflict effect compared to Risk (C > R, p < 0.005) | | | | | | | |
| 3 | | Left Middle Frontal Gyrus **( + )** | 10 | -31 | 53 | 7 | 1413 |
| 4 | | Left Middle Frontal Gyrus **( + )** | 6 | -26 | -7 | 57 | 875 |
| 5 | | Right Superior Frontal Gyrus **( + )** | 10 | 34 | 55 | 11 | 544 |
| 6 | | Left Precentral Gyrus **( + )** | 6 | -43 | 3 | 33 | 1656 |
| 7 | | Left Hypothalamus **( + )** |  | -8 | -6 | -6 | 141 |
| 8 | | Right Putamen **( + )** |  | 29 | -20 | -1 | 1604 |
| 9 | | Right Thalamus **( + )** |  | 19 | -24 | 6 | 2865 |
| 10 | | Left Thalamus **( + )** |  | -20 | -28 | 2 | 1142 |
| 11 | | Left Cingulate Gyrus **( + )** | 23 | 0 | -31 | 29 | 1483 |
| 12 | | Right Temporal Lobe **( + )** |  | 42 | -36 | -1 | 1055 |
| 13 | | Left Culmen **( + )** |  | -23 | -28 | -23 | 777 |
| 14 | | Right Parietal Lobe **( + )** | 7 | 23 | -66 | 33 | 36117 |
| 15 | | Left Parietal Lobe **( + )** |  | -13 | -66 | 39 | 40400 |
| 16 | | Right Occipital Lobe **( + )** | 19 | 24 | -67 | -3 | 29851 |
| 17 | | Left Occipital Lobe **( + )** | 19 | -24 | -67 | 1 | 28488 |
| Conflict effect compared to Ambiguity (C > A, p < 0.005) | | | | | | | |
| 1 | | Left Putamen **( + )** |  | -12 | 11 | -5 | 20 |
| 2 | | Right Inferior Frontal Gyrus **( + )** | 9 | 45 | 6 | 24 | 115 |
| 3 | | Left Parahippocampal Gyrus **( + )** | 37 | -26 | -46 | -8 | 122 |
| 4 | | Left Culmen **( + )** |  | -10 | -43 | -6 | 172 |
| 5 | | Right Parahippocampal Gyrus **( + )** | 19 | 20 | -48 | -2 | 784 |
| **#** | | **ROI** | **BA** | **x** | **y** | **z** | **size** |
| Conflict effect compared to Ambiguity (C > A, p < 0.005) | | | | | | | |
| 6 | | Right Parietal Lobe **( + )** | 7 | 3 | -70 | 51 | 174 |
| 7 | | Left Parietal Lobe **( + )** | 7 | -4 | -62 | 62 | 157 |
| 8 | | Left Parietal Lobe **( + )** | 7 | -15 | -68 | 51 | 583 |
| 9 | | Right Occipital Lobe **( + )** | 19 | 29 | -82 | 18 | 649 |
| 10 | | Right Occipital Lobe **( + )** | 18 | 11 | -73 | -6 | 228 |
| 11 | | Left Occipital Lobe **( + )** | 19 | -19 | -63 | -1 | 1198 |
| 12 | | Left Occipital Lobe **( + )** | 31 | -22 | -77 | 26 | 552 |
| 13 | | Left Cerebellum **( + )** |  | -9 | -77 | -15 | 289 |
| 14 | | Left Cerebellum **( + )** |  | -6 | -64 | -17 | 225 |
| 15 | | Left Cerebellum **( + )** |  | -17 | -60 | -11 | 201 |
| Ambiguity effect compared to Risk (A > R) correlated with ambiguity aversion  (Random Effects ANCOVA, p< 0.05) | | | | | | | |
| 1 | | Right Medial Frontal Gyrus **( + )** | 10 | 1 | 51 | 15 | 4765 |
| 2 | | Left Medial Frontal Gyrus **( + )** | 8 | 0 | 46 | 44 | 280 |
| 3 | | Right Superior Frontal Gyrus **( + )** | 8 | 21 | 41 | 42 | 1132 |
| 4 | | Right Superior Frontal Gyrus **( + )** | 6 | 16 | 7 | 60 | 287 |
| 5 | | Right Middle Frontal Gyrus **( + )** | 6 | 41 | 10 | 48 | 40 |
| 6 | | Left Middle Frontal Gyrus **( + )** | 8 | -22 | 32 | 42 | 1176 |
| 7 | | Left Amygdala **( - )** |  | -21 | -8 | -12 | 373 |
| 8 | | Left Insula **( - )** | 13 | -41 | -14 | 10 | 733 |
| 9 | | Right Caudate  **( - )** |  | 34 | -23 | -4 | 336 |
| **#** | | **ROI** | **BA** | **x** | **y** | **z** | **size** |
| Ambiguity effect compared to Risk (A > R) correlated with ambiguity aversion  (Random Effects ANCOVA, p< 0.05) | | | | | | | |
| 10 | | Right Striatum **( - )** |  | 20 | -31 | 25 | 305 |
| 11 | | Left Striatum **( - )** |  | -14 | -14 | 28 | 331 |
| 12 | | Right Cingulate Gyrus **( + )** | 31 | 6 | -37 | 38 | 785 |
| 13 | | Right Middle Temporal Gyrus **( + )** | 39 | 42 | -59 | 24 | 1132 |
| Conflict effect compared to Risk (C > R) correlated with conflict aversion  (Random Effects ANCOVA, p< 0.05) | | | | | | | |
| 1 | | Right Middle Frontal Gyrus **( + )** | 10 | 42 | 54 | 10 | 429 |
| 2 | | Right Middle Frontal Gyrus **( + )** | 6 | 23 | 19 | 54 | 697 |
| 3 | | Left Middle Frontal Gyrus **( + )** | 6 | -25 | 17 | 54 | 435 |
| 4 | | Left Middle Frontal Gyrus **( + )** | 6 | -22 | -6 | 59 | 1842 |
| 5 | | Left Inferior Frontal Gyrus **( + )** | 47 | -42 | 31 | -3 | 493 |
| 6 | | Left Inferior Frontal Gyrus **( + )** | 9 | -49 | 8 | 26 | 1079 |
| 7 | | Right Striatum **( - )** |  | 11 | 16 | 8 | 2167 |
| 8 | | Left Cingulate Gyrus **( - )** | 24 | -11 | 3 | 29 | 657 |
| 9 | | Right Parietal Lobe **( + )** | 40 | 36 | -36 | 53 | 7907 |
| 10 | | Paracentral Lobule **( + )** | 5 | 2 | -30 | 53 | 524 |
| 11 | | Left Superior Temporal Gyrus **( - )** | 22 | -30 | -49 | 15 | 1563 |
| 12 | | Right Inferior Temporal Gyrus **( + )** | 20 | 51 | -48 | -14 | 528 |

**Note: ( - )** – negative parameter estimates, **( + )** – positive parameter estimates.

**References:**

Bach DR, Seymour B, Dolan RJ (2009) Neural activity associated with the passive prediction of ambiguity and risk for aversive events. The Journal of Neuroscience 29:1648-1656.

Congdon P (2003) The basis for, and advantages of, Bayesian model estimation via repeated sampling. Applied Bayesian Modelling 1-30.

Hsu M, Bhatt M, Adolphs R, Tranel D, Camerer CF (2005) Neural Systems Responding to Degrees of Uncertainty in Human Decision-Making. Science 310:1680-1683.

Huettel S, Stowe CJ, Gordon E, Warner B, Platt M (2006) Neural Signatures of Economic Preferences for Risk and Ambiguity. Neuron 49:765-775.

Rustichini A, Dickhaut J, Ghirarda P, Smith K, Pardoe JV (2005) A brain imaging study of the choice procedure. Games and Economic Behavior 52:257-282.

Lancaster JL, Woldorff MG, Parsons LM, Liotti M, Freitas CS, Rainey L, Kochunov PV, Nickerson D, Mikiten SA, Fox PT, "Automated Talairach Atlas labels for functional brain mapping". Human Brain Mapping 10:120-131, 2000. [pdf] [PubMed]

Lancaster JL, Rainey LH, Summerlin JL, Freitas CS, Fox PT, Evans AC, Toga AW, Mazziotta JC. Automated labeling of the human brain: A preliminary report on the development and evaluation of a forward-transform method. Hum Brain Mapp 5, 238-242, 1997. [pdf]
